# The genetic structure of a *Brachypodium hybridum* population in a patchy arid landscape is independent of neighboring perennials and stable over two consecutive years

**DOI:** 10.7717/peerj.20787

**Published:** 2026-03-02

**Authors:** Megan K. Korte, Louis van de Zande, Antonio J. Manzaneda, Marco van der Velde, Rampal S. Etienne, Christian Smit

**Affiliations:** 1 Groningen Institute for Evoutionary Life Sciences, University of Groningen, Groningen, Netherlands; 2 Center for Conservation Biology, University of California, Riverside, Palm Desert, CA, United States of America; 3 Departamento de Biología Animal, Biología Vegetal y Ecología, Universidad de Jaén, Jaén, Spain

**Keywords:** *Brachypodium hybridum*, Population genetics, Facilitation, Local adaptation, Arid

## Abstract

In arid landscapes perennials can provide protection for annual plants against environmental stress such as drought. This could lead to population sub-structuring of the annuals, because of position dependent reproductive success, and/or competition for association with perennial shrubs. Although such interactions are well known drivers of plant community dynamics, little is known about the local and temporal effects of perennial presence on the genetic structure of annuals. As a first step to address this, we used a set of microsatellite markers to assess the genetic structure of the annual grass *Brachypodium hybridum* (L.) P. Beauv. growing underneath and outside the canopy cover of perennials at an arid site in southern Spain over two consecutive years (2018 and 2019). Upon sampling, we observed clear but inconsistent seasonal differences in phenology between individuals underneath or outside canopies. However, we did not find genetic differentiation between sampling location or sampling year (four populations: overall *F*_ST_ = 0.024). An analysis of the overall genetic structure inferred three putative clusters, but these clusters were neither associated with location nor with year of sampling. We conclude that the genetic structure of this *B. hybridum* population is independent of neighboring perennials and stable over two consecutive years and is not associated with phenological differences. The data further show a high level of homozygosity in the population, and the recurrent presence of identical genotypes, both with respect to location and year, indicating a major role of selfing in *B. hybridum* reproduction. However, the samples collected outside the canopies in 2019 show a slightly higher value of estimated heterozygosity. This may indicate the effect of immigration from other *B. hybridum* populations. These results show that neighboring perennial plants do not affect the stable genetic structure of the *B. hybridum* population within this arid site, but that other factors such as immigration, could eventually result in local and temporal differences. These results also indicate a possible patchy distribution of identical genotypes of the annuals, which should be considered when sampling such populations.

## Introduction

The canopy cover of perennial plants may provide protection for annual plants against prevailing stressful environments (Berdugo et al., 2019; Bruno, Stachowicz & Bertness, 2003; Tilman, 1982). This positive effect of the perennial plant, termed facilitation, could lead to differences in fitness related traits, such as flower production, flowering time, and fruit production, between subpopulations underneath and outside canopy cover (Halliday, Koornneef & Whitelam, 1994; Tirado & Pugnaire, 2003). Indeed, it was found that shrub presence positively affected the biomass and survival of two different ecotypes of *Brachypodium distachyon* (L.) P. Beauv in an arid landscape (Liancourt & Tielbörger, 2011). Alternatively, the beneficial properties of the microhabitat as provided by the canopy of perennials, such as higher levels of soil moisture and lower air temperatures (Hannah et al., 2014; Lortie et al., 2022; Maestre et al., 2001; Morelli et al., 2017) may vary temporally. Aggregation of individuals in the buffered microhabitat, may lead to competition for limited resources amongst the benefactors (Luzuriaga et al., 2012). In addition, as stress increases further (*e.g.*, during extensive droughts), the positive interactions between the perennial plant and the understory may weaken or even collapse (Michalet et al., 2014). Both facilitation and competition may generate different levels of selection by favoring different fitness traits based on the spatial association to the perennial plant (Richardson et al., 2014; Verdú et al., 2021). Several studies have demonstrated that fitness related traits such as flowering time (Halliday, Koornneef & Whitelam, 1994; Tirado & Pugnaire, 2003), fruit production (Tielbörger & Kadmon, 1995) and germination rates (O’Brien et al., 2021) can differ between subpopulations underneath and outside canopy cover. While such facilitation effects can affect phenotypic evolution (Verdú et al., 2021), empirical tests of whether facilitation produces within-population genetic differentiation are scarce. A study by Castellanos et al. (2014) found that facilitation homogenized genetic variation rather than drove divergence within populations of *Euphorbia nicaeensis*, despite differences in phenology.

The *Brachypodium distachyon* complex consists of three annual grass species that are native to the Mediterranean Basin, occur in semi-arid regions with varying degrees of environmental stress and experience pronounced seasonality. The diploids *B. distachyon* (2n = 10) and *B. stacei* Catalán, Joch. Müll. & Hasterok (2n = 20) and the allotetraploid *B. hybridum* (2n = 30), which is a hybrid between *B. distachyon* and *B. stacei* (Catalán et al., 2012). The parental subgenomes of *B. hybridum* have been shown to have high subgenomic stability (Mu et al., 2023), with no signs of homologous recombination (Scarlett et al., 2022). Although several species in the *Brachypodium* genus are rhizomatous, there is currently no evidence to suggest clonal reproduction for either of these three species. They occur in semi-arid regions with varying degrees of environmental stress and experience pronounced seasonality (Chesson et al., 2004). The *Brachypodium distachyon* complex shows large environmental variation in life history characters, including flowering time (Schwartz et al., 2010), vernalization requirements (Woods et al., 2017), drought tolerance (Manzaneda et al., 2015) and plant height (Tyler et al., 2014). Several studies have reported a large variety in phenotypic traits (Manzaneda et al., 2015) and a relatively high genetic diversity between *Brachypodium* populations (Marques et al., 2017; Neji et al., 2015; Shiposha et al., 2016). Although several studies also have found high levels of genetic diversity within populations (Dell’Acqua et al., 2014; Neji et al., 2015; Vogel et al., 2009), we are unaware of any studies that have investigated genetic diversity within *Brachypodium* populations, considering the presence of neighboring perennial plants. Similarly, there is little information on the relative contribution of reproduction by residents and migration from other sites to the temporal population structure of annual grasses.

In order to understand the ecological dynamics of annual species in arid landscapes with perennial vegetation, it is crucial to disentangle temporal and microclimate effects on their genetic structure. Hence, as a first step to gain insight into the local and temporal dynamics of an annual grass species in an arid landscape, we assessed whether the genetic variation of *B. hybridum* collected underneath and outside perennial plant canopy cover differs over two consecutive years.

## Materials & Methods

### Study site

**Figure 1:**
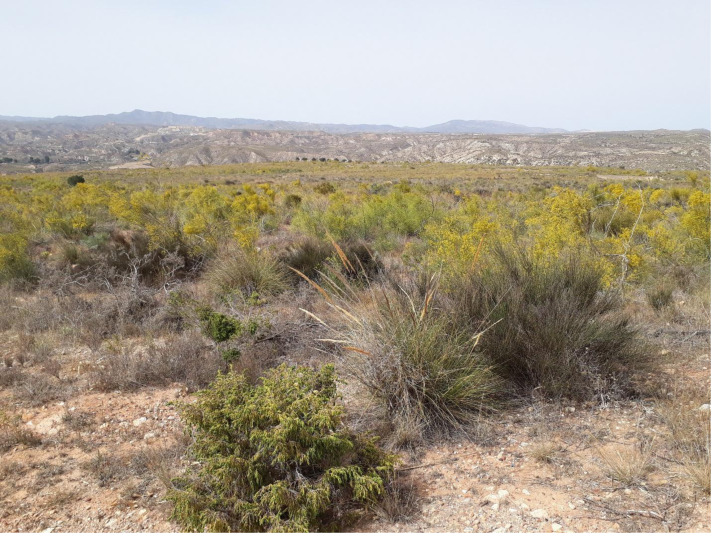
Site of population sampled. Photo Credit: Megan Korte.

The study site was located in the Almeria province, four km east of the city of Sorbas, in southeastern Spain (latitude 37°05′43.00″ longitude 2°5′7.00″). This site was the most arid site used in a previous study in which we found morphological differences between *B. hybridum* found underneath in contrast to outside neighboring perennial plant cover (Korte et al., 2025). The site is considered arid with regard to the Global Aridity Index (GAI) of CGIAR-CSI (http://www.cgiar-csi.org; (Zomer et al., 2007; Zomer et al., 2008)) a global database that contains mean monthly precipitation and potential evapotranspiration collected from 1950–2000, which we extracted from this database for this site. The average annual precipitation is 282 mm/year. The 30 year mean annual temperature is 16.6 °C mean average maximum and minimum temperatures are 2.6 °C in January and as high as 30.8 °C in August. This site has an elevation of 387 m and a 9% northerly facing slope and is approximately 0.5 hectares in size. The plant community consists of a patchwork of Mediterranean woody shrubs, perennial bunchgrasses and sparsely vegetated open areas dominated by spring annuals and perennials and is approximately one-half hectare in size (Fig. 1). Within the site, *Brachypodium* co-occurs with perennial plants ranging from 0.5–5.0 m in height that provides a partially shaded understory throughout the day. The dominant perennial plant species in this area are the leguminous shrub *Retama sphaerocarpa* and the common bunchgrass *Stipa tenacissima*.

### Sampling

**Figure 2:**
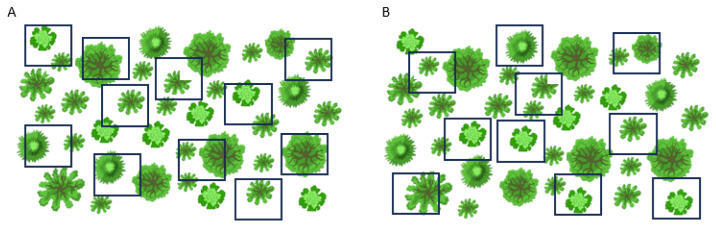
Diagram illustrating stratified random sampling across two sampling periods. (A) and (B) reflect plot layouts per sampling period. Although the placement of plots were not identical for each sampling, full site coverage did occur. *Shrub* icons adapted from ONYXprj, *via* iStock (Asset ID 1485414312), used under Standard License.

We used a stratified random design to sample this site four times: Spring 2018 (in/out), Fall 2018 (in), Spring 2019 (out) and Fall 2019 (in/out). Samples were labeled based on their position (“in” or “out”) and sampling season. Sampling had to be conducted in both spring and fall because viable tissue was not consistently available across canopy positions in every season due to phenological constraints. Sampling from seasons within the same year were combined to obtain single yearly samples of either “in” or “out” (see Table S1). Sampling of *Brachypodium* underneath or outside perennial canopy cover was conducted in 10–15 plots (10 × 10 m each) throughout the site during every sampling period (Fig. 2). Each plot contained several perennial plants, and each *Brachypodium* plant was collected from underneath the canopy cover of each perennial plant within the plot. To minimize the likelihood of sampling plants with the same maternal origin, plant samples were collected at least one meter apart throughout the site during each sampling period (Manzaneda et al., 2012). As *Brachypodium* was not consistently found under every shrub, a few plots were revisited later in the same sampling day. In these cases, we avoided previously sampled points and maintained the >1 m spacing rule so that no deliberate resampling of the same plant or sampling point occurred.

### DNA extraction and species discrimination

We stored the plant samples at −20 °C until analysis. Leaves and stems of field collected plants with weights between 0.01–0.10 g were added to a tube containing a four mm steel ball, snap frozen in liquid nitrogen and then pulverized with a Mixer Mill (Retsch MM400). DNA was extracted using a modified CTAB buffer stock solution which was composed of 2% w/v CTAB, 5M NaCl, 0.5M EDTA, 1M TrisHCl pH 8.0, 2% w/v polyvinylpyrrolidone-40 (Doyle, 1991; Doyle & Doyle, 1987). DNA concentration and purity were measured with a Thermo Scientific NanoDrop 2000.

To test whether DNA extraction failures caused a sampling bias, we compared the distribution of samples that failed DNA extraction between “in” and “out” locations and between 2018 and 2019 collections. Counts were summarized in a 2 × 2 contingency table (Year × Location), and a Fisher’s exact test was used to evaluate independence between categories. Analyses were conducted in R v.4.4.0 (R Core Team, 2024).

Species discrimination between *B. distachyon* and *B. hybridum* is possible by amplifying a diagnostic insertion/deletion in the promoter region of the *BdALMT1* gene. The species was considered *B. distachyon* if only a 506 bp fragment was amplified, and *B. hybridum* if both a 506 bp fragment and a 629 bp fragment (from the *B. stacei* subgenome) were amplified (Contreras et al., 2017).

The amplification reaction to identify the species was performed in Taq PCR 2x Master Mix (Qiagen) (6 µL), DNA 3.2 µL (10 ng/ µL), and 1.2 µL (5 p moles) each of the forward primer (5′-CCGAATACACATCGACCTCCTCAT-3′) and the reverse primer (5′-CCCGAGCCCGAGGACCAACGAG-3′) and distilled water for a final volume of 12 µL. The PCR reaction was conducted with an Eppendorf MasterCycler nexus thermocycler with an initial denaturation step at 95 °C (3 min), followed up by 35 cycles of 95 °C (20 s), 55 °C (30 s) and 72 °C (1 min) and the final extension at 72 °C (7 min). Amplification products were separated by electrophoresis in a 2.0% TBE gel. The reference accessions Bd21 or Bd30 were used as a size reference control (Contreras et al., 2017).

To assess the genotypic and allelic variation of this population of *B. hybridum*, we tested 17 primer sets developed for the amplification of microsatellite loci of the diploid species *B. distachyon*: ALB006, ALB008, ALB034, ALB040, ALB056, ALB086, ALB087, ALB089, ALB100, ALB131, ALB139, ALB158, ALB165, ALB230, ALB256, ALB372 and ALB445 (Vogel et al., 2009) (Table 1). As *B. hybridum* is an allotetraploid, collectively the primer sets target loci on both the *B. distachyon* subgenome, and loci on the *B. stacei* subgenome (Gordon et al., 2020; Goodstein et al., 2012). In our hands, the 17 primer sets yielded 23 amplified loci, across both subgenomes (D + S). After excluding five monomorphic loci and loci with unreliable amplification, 18 informative loci remained and were used for analyses (Table 1).

**Table 1:** Microsatellite loci evaluated for genotyping. Each primer set is listed with its corresponding subgenome (D or S), chromosomal location (in megabases) and whether it was used in the final genotyping analysis. Loci were excluded if they were monomorphic or showed unreliable amplification. Chromosome locations are based on the *B. hybridum* (v1.1) reference genome (source: https://phytozome-next.jgi.doe.gov/).

**Primer set**	**Locus name**	**Subgenome**	**Chromosome**	**Position (Mb)**	**Included in final analysis**	**Exclusion reason**
1	ALB006	D	4	46.5	Yes	
	ALB006	S	5	22	Yes	
2	ALB008	D	4	31.7	Yes	
	ALB008	S	5	–	No	Monomorphic
3	ALB034	D	1	13.1	Yes	
4	ALB040	D	3	1.1	Yes	
5	ALB056	D	2	45.9	Yes	
	ALB056	S	1	12.6	Yes	
6	ALB086	D	2	3.7	Yes	
7	ALB087	D	1	9.5	Yes	
8	ALB089	D	4	5.4	Yes	
9	ALB100	D	1	73	Yes	
	ALB100	S	9	8.5	Yes	
10	ALB131	D	4	42.3	Yes	
11	ALB139	D	5	–	No	Unreliable amplification
	ALB139	S	–	–	No	Monomorphic
12	ALB158	D	2	34.1	Yes	
13	ALB165	D	1	35.6	Yes	
	ALB165	S	–	–	No	Monomorphic
14	ALB230	D	2	–	No	Monomorphic
15	ALB256	D	1	41.5	Yes	
16	ALB372	D	1	47.6	Yes	
17	ALB445	D	3	35.6	Yes	
Total = 17					Total analyzed = 18	Total amplified = 23

Specific forward primer sequences were all labeled with the universal M13 sequence (5′-CACGACGTTGTAAAACGAC-3), to enable labeling of the amplicons for analysis (Boutin-Ganache et al., 2001) (see below). The PCRs were carried out in a final volume of 20 µl in an Eppendorf MasterCycler thermocycler with a thermal profile consisting of a 2-min initial denaturation step at 95 °C followed by 20 s at 95 °C, 20 s at 54 °C (4 microsatellite markers at 50 °C) and 1 min at 72 °C for 35 cycles. Reactions were carried out in 1x Buffer, of 2.5 mM MgCl_2_, 0.25 mM dNTPs and 1.0 U *Taq* Polymerase Promega, 100 ng genomic DNA, 0.25 µM marker-specific reverse primer, 0.25 µM marker-specific M13-tailed forward primer and 0.5 µM fluorescent-labeled (FAM or HEX) universal M13 primer (5′-CACGACGTTGTAAAACGAC-3′). PCR products were separated on a AB3730 DNA analyzer and allele sizes were scored using Gene Mapper V4.0 software (Applied Biosystems) based on the internal Genescan-500 ROX size standard (Applied Biosystems) (Table S2).

### Genetic analysis

We used the program FSTAT 2.9.3.2 (Goudet, 1995) to calculate the observed heterozygosity (H_o_) and expected heterozygosity (H_e_), estimates of departure from random mating (*F*_IS_) and genetic differentiation (Weir & Cockerham, 1984). Standard errors for *F*_IS_ and *F*_ST_ were estimated by jackknifing over loci. Significance of pairwise *F*_ST_ estimates were tested in FSTAT using 120 random permutations per comparison with correction for multiple testing; with adjusted *p* <  0.05 were considered significant. We calculated the selfing rate *s* as (2*F*_IS_/(1 + *F*_IS_)) (Ritland, 1990).

We used an analysis of molecular variance (AMOVA) to test for hierarchical genetic structure among canopy positions and sampling years using the poppr package in R (Kamvar, Tabima & Grünwald, 2014). AMOVA requires complete datasets with no missing genotypes; therefore, individuals or loci containing missing genotypes were excluded, reducing the dataset from 173 to 115 individuals. Individuals were grouped by canopy position (“in”, *n* = 57; “out”, *n* = 58) and by sampling year (2018, *n* = 60; 2019, *n* = 55). We used 999 random permutations to assess the significance of variance components and associated Φ-statistics (analogous to Wright’s F-statistics). Separate AMOVAs were run for canopy position (“in” *vs* “out”) and sampling year (2018 *vs* 2019) (Table S3).

For analyzing the genetic structure we used the program Structure (version 2.3; (Pritchard, Stephens & Donnelly, 2000) The settings were: admixture model, 20000 burn-in and 20000 Markov Chain Monte Carlo (MCMC) length, allele frequencies uncorrelated, no prior location/population assignment. The optimal number of putative clusters *K* was derived from the calculated posterior likelihood L(K) and its second order derivative Δ(*K*) (Evanno, Regnaut & Goudet, 2005) using 20 independent runs for each *K*.

## Results

### Samples

We collected a total of 690 specimens (305 under perennial canopy, 385 outside canopy). A total of 451 samples from too small or too senesced plants, failed DNA extraction (2018: 202; 2019: 249), distributed among 72 “in” and 130 “out” samples in 2018, and 97 “in” and 152 “out” samples in 2019. From the 239 remaining samples, 66 were identified as *B. distachyon* and excluded because their numbers were too small for meaningful comparison, leaving 173 *B. hybridum* samples for final analysis. The 173 genotyped *B. hybridum* samples comprised of 84 collected under perennial cover and 89 collected outside perennial cover. Although these numbers are in balance, samples inconsistently varied over location and season (Table S1). Sampling in spring 2018 overall yielded more material than sampling in fall 2018. Inversely, sampling in spring 2019 overall yielded less material than sampling in fall 2019. Interestingly, no “out” samples were obtained in fall 2018, while no “in” samples were obtained in spring 2019. This was largely due to the phenology of the plants, prohibiting high quality DNA extraction. To summarize: sample sizes varied between sampling years owing to differential quality: for 2018 we used 51 “in” and 33 “out” samples, and for 2019 we used 33 “in” and 56 “out” samples. For genotyping analysis, we divided the samples over four categories according to location and year: 2 for 2018 (51 “in” samples/33 “out” samples), and 2 for 2019 (33 “in” samples/56 “out” samples). This asymmetry was not due to sampling bias but reflected real phenological differences in plant development across canopy positions and years, likely shaped by winter conditions.

### DNA extraction bias testing

We used a Fisher’s exact test to test if there was a bias with DNA extraction with regard to our sampling. We found that there was not a significant association between extraction success and either sampling location or year (*p* = 0.49; odds ratio = 0.86; 95% CI [0.57–1.29]). These results confirm that DNA extraction failures occurred randomly with respect to site and year, and thus, did not bias the final genotyped dataset.

### SSR genotyping

**Figure 3:**
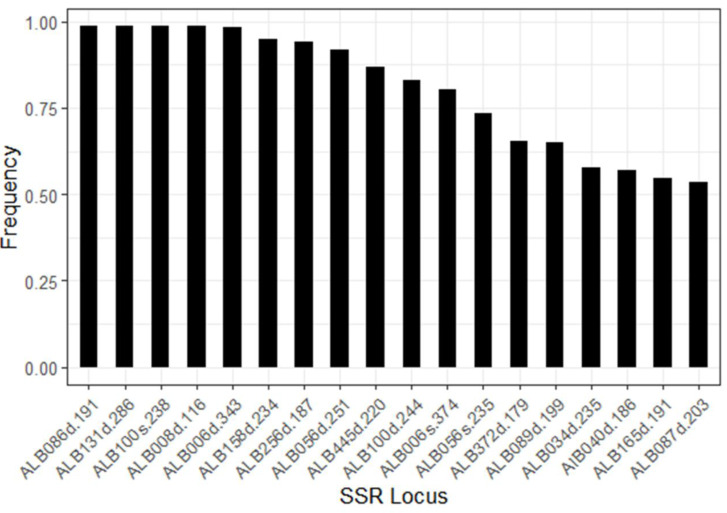
Frequency of the most common allele for each of the 18 analyzed SSR loci. For each locus, the most frequent allele was identified and its proportion calculated across the full dataset (*n* = 173). Locus codes reflect primer set and subgenome origin: suffix (D) indicates amplification from the *B. distachyon* subgenome, and (S) from the *B. stacei* subgenome.

We tested 17 SSR (Simple Sequence Repeat) primer sets. Six primer sets amplified loci from both subgenomes (ALB006, ALB008, ALB056, ALB100, ALB139, ALB165), and these were treated as independently segregating, resulting in 23 amplified loci. Four of these 23 loci were monomorphic (ALB008S, ALB230D, ALB165S, ALB139S) and one (ALB139D) produced unreliable amplification. Therefore, five loci were excluded from further analysis, leaving 18 informative SSR loci for downstream analyses (Table 1). The average number of alleles per locus was 3.5, ranging from two to six. The allele frequencies ranged from 0.01 to 0.99 with an average of 0.29. The most frequent alleles (per locus) had frequencies ranging from 0.54 to 0.99 (Fig. 3). Based on these data, the expected heterozygosity (H_e_) for the entire sample set was calculated to be 0.27. The observed heterozygosity (H_o_), however, was 0.01, which is very low in comparison to H_e_ (Table 2). A re-analysis, using only the 50% markers with the lowest value for the most frequent allele, yielded a H_e_ of 0.47. The H_o_, however, remained very low with a value of 0.02. This indicates that the low H_o_ is not a result of the allele frequencies of the loci used, but a reflection of the high degree of selfing as a reproductive mode of *B. hybridum*. This is also reflected in the value of *F*_IS_ for the whole dataset that was calculated to be 0.96 (SE 0.04).

No genetic differentiation was observed when analyzing the whole dataset divided into the four sampling categories (*F*_ST_ = 0.024; SE 0.007). In agreement with this, no genetic differentiation was observed between all samples collected outside (H_e_ = 0.29) or underneath (H_e_ = 0.26) (Table 2) the canopy cover; *F*_ST_ = 0.005 (SE 0.004). Similar values were obtained when comparing samples originating outside or underneath canopy cover for 2018 (*F*_ST_ = 0.008; SE 0.01) and 2019 (*F*_ST_ = 0.052; SE 0.017) (Table 3). Significance of pairwise *F*_ST_ estimates were tested in FSTAT using 120 random permutations per comparison with correction for multiple testing; no pairwise comparison was significant *p* > 0.05. The AMOVA results support the *F*_ST_ findings, showing that most genetic variation occurred among individuals within groups (>96%) (Table S3). Only 0.6% of the variance was explained by canopy position (ΦLoc-Total = 0.0057, *p* = 0.22) and less than 0.2% by sampling year (ΦYear-Total = −0.0018, *p* = 0.44), indicating no significant difference by either factor.

**Figure 4:**
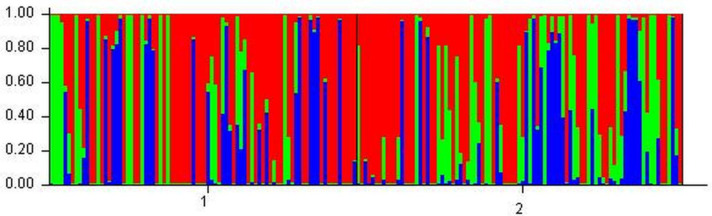
Genetic clustering of *B. hybridum* individuals between canopy positions. Distribution of individual genotypes between canopy positions (1 = In and 2 = Out). Each vertical bar represents one individual, partitioned into colored segments corresponding to putative genetic clusters.

**Figure 5:**
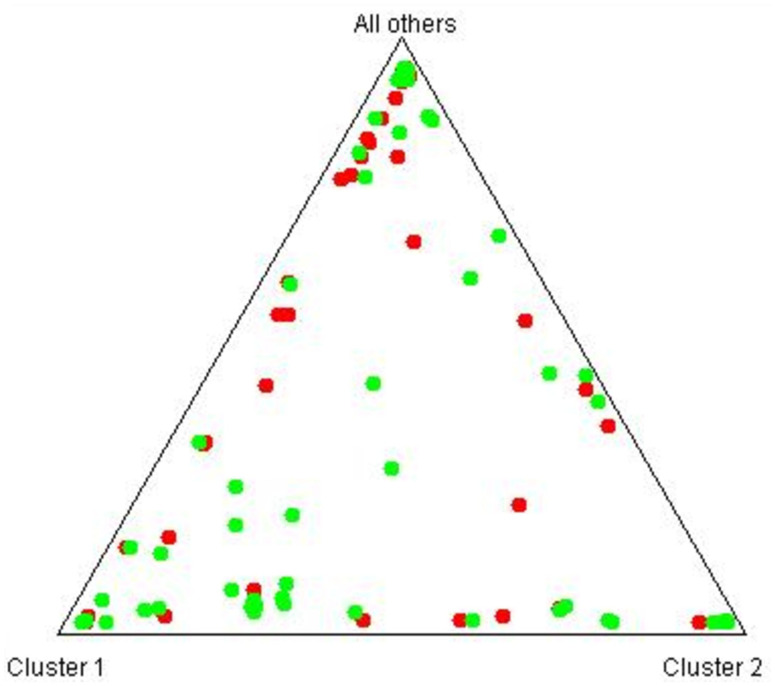
Triangle plot showing assignment of *B. hybridum* individuals to genetic clusters. Colors indicate canopy position (red = In, green = Out).

These results show that shelter provided by perennials did not lead to genetic differentiation of the annual *B. hybridum* population at this site. The analysis of the genetic structure, with 20 runs for seven populations resulted in three putative genetic clusters (*K*) (Fig. 4), with Δ(*K*) = 49 being substantially larger than Δ(*K*)-values for other *K* values (Fig. 5). However, individuals from the four sampling categories did not aggregate into any of these clusters but were evenly dispersed over all three. This indicates that the distribution of genotypes is neither dependent on canopy cover nor on year of sampling. Analyzing the genetic structure using unique genotypes only, still led to the inference of three clusters, but with Δ(*K*) = 17. Genotypes were evenly distributed over these clusters (Fig. S1).

**Table 2:** Expected (He) and observed (Ho) heterozygosity by year and canopy position (In *vs* Out).

Year/Location	He	Ho
All	0.27	0.01
All - In	0.26	0.01
2018 In	0.26	0.01
2019 In	0.24	0.01
All - Out	0.29	0.01
2018 Out	0.20	<0.01
2019 Out	0.32	0.01
2018 In/Out	0.24	0.01
2019 In/Out	0.30	0.01

**Table 3:** Pairwise genetic differentiation across canopy positions and sampling years. Genetic differentiation metrics (Theta F_ST_ = fixation index, F_IS_ = inbreeding coefficient, S = selfing rate, SE = standard error) across sampling periods. Significance of pairwise F_ST_ estimates was tested in FSTAT using 120 random permutations per comparison with correction for multiple testing; no pairwise comparison was significant (*p* > 0.05).

Sampling period	Theta (Fst)	SE	Fis	SE	S
2018 *vs* 2019 In & Out	0.004	0.005	0.965	0.007	0.982
2018 & 2019 In *vs* Out	0.005	0.004	0.964	0.007	0.982
2018 In *vs* Out	0.008	0.01	0.967	0.012	0.983
2019 In *vs* Out	0.052	0.017	0.962	0.01	0.981
2018 In *vs* 2019 In	0.013	0.011	0.965	0.01	0.982
2018 Out *vs* 2019 Out	0.053	0.013	0.963	0.009	0.981

The subset of samples for outside canopies 2019 had the highest H_e_ (0.32). Although this did not lead to a clear differentiation of the subsamples involved, it may be indicative of subtle differences in the *B. hybridum* population composition between 2018 and 2019 (Tables 2 and 3). In accordance with the high selfing rate, 14 multilocus genotypes were encountered more than once both over location and seasons, accounting for ∼50% of all genotypes. The most frequent multilocus genotype constituted of 22.5% of all genotypes determined; 30% in 2018 and 16% in 2019. The distribution of this genotype inside or outside canopy cover was comparable between 2018 (“in” 64%, “out” 36%; *n* = 25) and 2019 (“in” 57%, “out” 43%; *n* = 14). This again indicates the absence of clear population substructuring because of perennial plant presence. However, seven multilocus genotypes were observed more than once in 2019 only. They were found in 17 samples, 10 of which occurred outside and seven inside canopy cover. This subtle difference may possibly indicate immigration from outside the sampling area.

## Discussion

The aim of the current study was to gain insight into the local and temporal genetic structure of an annual grass species in an arid patchy landscape, in relation to neighboring perennials. We found that the presence of neighboring perennials did not affect the genetic structure of the *B. hybridum* population and that this genetic structure was stable over two consecutive years (2018 and 2019). Neither sampling locations nor year of sampling showed signs of genetic differentiation (overall *F*_ST_ = 0.024). Although an analysis by Structure is not appropriate to detect only one cluster, the likelihood plot seems to indicate that Δ*K* is probably highest for *K* = 3. However, the sampled individual genotypes did not aggregate into any of these putative clusters. This is in agreement with the conclusion that our samples are derived from a genetically non-differentiated population.

Interestingly, the two consecutive years did show variation in the phenology of the annuals. In spring 2018, more samples could be obtained than in fall 2018, where no “out” samples could be obtained. The reverse is true for 2019, where fall yielded more samples than spring, in which no “in” samples were obtained. It is not clear if this difference is caused by a stressful winter between 2018 and 2019, but it does show phenotypical variation across locations. Despite this asymmetry, we found no genetic differentiation between canopy positions suggesting that the microhabitat, as provided by neighboring perennial plants, may buffer environmental variation without driving divergence. This is in agreement with the conceptual study of Castellanos et al. (2014), who demonstrated a comparable independence of phenotypic plasticity and genetic variation in *Euphorbia nicaeensis*.

A very high level of homozygosity was observed, indicating that selfing is the predominant reproduction mode of *B. hybridum* in this population. Furthermore, identical genotypes, making up to ∼50% of all genotypes, were observed to be present both underneath and outside canopy cover and to persist over consecutive years. The occurrence of these genotypes was only subtly different in 2019 and may reflect immigration.

The genotypic diversity found in our study, is in agreement with the study that is the source of the applied SSR markers (Vogel et al., 2009). In addition, our results are similar to the results for the Iberian peninsula reported by Shiposha et al. (2020), who found that *B. hybridum* is highly selfing with evidence of previous migration events in only one sampling period. However, the genetic diversity (number of alleles per locus) of our study is different from that found by Neji et al. (2015), who considered nine populations of *B. hybridum* in Tunisia using 15 loci, 14 of which were in common with this study. While we found 87 unique alleles and 3.5 alleles per locus when combining the two subgenomes present, they found a relatively high number of unique alleles (156) and the average number of alleles per locus was much higher at 10.6. Apparently, genetic variation of regional *B. hybridum* populations can be quite different over larger geographical scales (Neji et al., 2015; Shiposha et al., 2020) and the population may even differ depending on environmental conditions (Hammami et al., 2014).

It should be noted that sampling various locations to determine population structure should consider the fact that identical genotypes may temporally be frequent in any locality. Unfortunately, our sampling method did not allow to assess whether these identical genotypes occur clustered or dispersed, but the fact that most of these identical genotypes were encountered both underneath and outside canopies indicates that they are not confined to small areas of a location. The chance of the most frequent identical genotype in our study to occur, based on allele frequencies of the whole dataset, is only 0.02%. Yet, this genotype was sampled 39 times (22.5% of all samples). This should be taken into account when sampling individual genotypes to represent a population of *B. hybridum*.

Overall, we found a high level of observed homozygosity, reflecting the fact that *B. hybridum* is a highly selfing species. This is in line with previous studies that report homozygous individuals to dominate *B. distachyon, B. stacei* and *B. hybridum* populations found along the Mediterranean basin (Neji et al., 2015; Shiposha et al., 2016; Vogel et al., 2009). Further, we did not find differences in the level of expected homozygosity for samples collected outside or under perennial canopy, apart from the outside canopy samples of 2019. An explanation for this relatively higher level of expected heterozygosity (H_e_ = 0.32) could be the occurrence of immigration by long distance dispersal. As *Brachypodium* has relatively large awns and hairy seeds which can attach to fur or feathers, dispersal by animals is a realistic possibility. Long distance dispersal by grazing animals has been reported (Couvreur et al., 2004) as well as by sheep herds in Spain (Manzano & Malo, 2006). Neji et al., (2015) support long distance dispersal events occurring as they could explain the high levels of genetic variation found in *B. hybridum* populations in Tunisia by assuming two migrants per generation. A more specialized design would be required to monitor if such events apply to our Spanish population as well.

## Conclusions

No genetic difference was found between samples from underneath or from outside perennial canopies. This indicates that the presence of perennials did not cause genetic differentiation between these groups with respect to the SSR markers. Therefore, possible genetic differences in traits controlling phenology, morphology or reproduction, driven by facilitation (Castellanos et al., 2014; Michalet et al., 2014; Schwartz et al., 2010) are not reflected in the level of homozygosity of these SSR markers. The microsatellites used here cover each of the five chromosomes, in which the larger chromosomes contain more markers of the *B. distachyon* subgenome. In addition, we found that six markers (each on a unique chromosome) were also represented from the *B. stacei* subgenome. We could successfully analyze three of these loci. As a result, we have resolving power to conclude that both the similar level of homozygosity and the low level of genetic differentiation indicate the absence of strong selection events. Such selection events would have been reflected in linkage disequilibrium of some markers with either advantageous or disadvantageous alleles of possible fitness related genes. Moreover, the absence of genetic differentiation for plants derived from underneath or outside perennial canopies is in agreement with the finding in an earlier study in which no differences in flowering time were detected within this arid site (Korte et al., 2025). Overall, we conclude that there is no evidence for genetic differences driven by facilitation in this population of *B. hybridum*. Any morphological differences between the subpopulations could be due to divergence in a few loci that are not closely linked to the marker genes used.

##  Supplemental Information

10.7717/peerj.20787/supp-1 Supplemental Information 1 Sampling summary by canopy position, season, and year Number of samples for each combination of canopy position (“in” = under perennial canopy; “out” = outside canopy) and sampling period.

10.7717/peerj.20787/supp-2 Supplemental Information 2 Alleles for the 23 amplified SSR loci

10.7717/peerj.20787/supp-3 Supplemental Information 3 Analysis of molecular variance (AMOVA) testing genetic differentiation of among canopy positions (“In” *vs* “Out”) and sampling years (2018 *vs* 2019) AMOVA showing partitioning of genetic variation between canopy locations (In *vs* Out), among individuals within locations, and within individuals. Reported columns: degrees of freedom (df), sum of squares (SS), estimated variance component (*σ*), percent of total variation (%), Φ-statistics for each hierarchical comparison. *P*-values based on 999 permutations; *** denotes *p* < 0.001.

10.7717/peerj.20787/supp-4 Supplemental Information 4 Genetic structure of four sampling categories of *B. hybridum* analyzed with 18 SSR markers. (A) and (B) Estimated Ln probability of data (L(K)) and Δ (K), using 20 independent runs for each K

## Additional Information and Declarations

### Data Availability

The following information was supplied regarding data availability: The raw data for all allele sizes for all 18 loci used is available in the Supplementary File.

## References

[ref-1] Berdugo M, Maestre FT, Kéfi S, Gross N, Le Bagousse-Pinguet Y, Soliveres S (2019). Aridity preferences alter the relative importance of abiotic and biotic drivers on plant species abundance in global drylands. Journal of Ecology.

[ref-2] Boutin-Ganache I, Raposo M, Raymond M, Deschepper CF (2001). M13-tailed primers improve the readability and usability of microsatellite analyses performed with two different allele-sizing methods. Biotechniques.

[ref-3] Bruno JF, Stachowicz JJ, Bertness MD (2003). Inclusion of facilitation into ecological theory. Trends in Ecology & Evolution.

[ref-4] Castellanos MC, Donat-Caerols S, González-Martínez SC, Verdú M (2014). Can facilitation influence the spatial genetics of the beneficiary plant population?. Journal of Ecology.

[ref-5] Catalán P, Müller J, Hasterok R, Jenkins G, Luis Mur AJ, Langdon T, Betekhtin A, Siwinska D, Pimentel M, López-Alvarez D (2012). Evolution and taxonomic split of the model grass *Brachypodium distachyon*. Annals of Botany.

[ref-6] Chesson P, Renate Gebauer LE, Schwinning S, Huntly N, Wiegand K, Morgan Ernest SK, Sher A, Novoplansky A, Weltzin JF (2004). Resource pulses, species interactions, and diversity maintenance in arid and semi-arid environments. Oecologia.

[ref-7] Contreras R, Figueiras AM, Gallego FJ, Benavente E, Manzaneda AJ, Benito C (2017). Neutral molecular markers support common origin of aluminium tolerance in three congeneric grass species growing in acidic soils. AoB Plants.

[ref-8] Couvreur M, Vandenberghe B, Verheyen K, Hermy M (2004). An experimental assessment of seed adhesivity on animal furs. Seed Science Research.

[ref-9] Dell’Acqua M, Zuccolo A, Tuna M, Gianfranceschi L, Pè ME (2014). Targeting environmental adaptation in the monocot model *Brachypodium distachyon*: a multi-faceted approach. BMC Genomics.

[ref-10] Doyle J, Hewitt GM, Johnston AWB, Young JPW (1991). DNA protocols for plants. Molecular techniques in taxonomy.

[ref-11] Doyle JJ, Doyle JL (1987). A rapid DNA isolation procedure for small quantities of fresh leaf tissue. Phtochemical Bulletin.

[ref-12] Evanno G, Regnaut S, Goudet J (2005). Detecting the number of clusters of individuals using the software STRUCTURE: a simulation study. Molecular Ecology.

[ref-13] Goodstein DM, Shu S, Howson R, Neupane R, Hayes RD, Fazo J, Mitros T, Dirks W, Hellsten U, Putnam N (2012). Phytozome: a comparative platform for green plant genomics. Nucleic Acids Research.

[ref-14] Gordon SP, Contreras-Moreira B, Levy JJ, Djamei A, Czedik-Eysenberg A, Tartaglio VS, Session A, Martin J, Cartwright A, Katz A (2020). Gradual polyploid genome evolution revealed by pan-genomic analysis of *Brachypodium hybridum* and its diploid progenitors. Nature Communications.

[ref-15] Goudet, JFSTAT (1995). FSTAT (version 1.2): a computer program to calculate F-statistics. Journal of Heredity.

[ref-16] Halliday KJ, Koornneef M, Whitelam GC (1994). Phytochrome B and at least one other phytochrome mediate the accelerated flowering response of *Arabidopsis thaliana* L. to low red/far-red ratio. Plant Physiology.

[ref-17] Hammami R, Jouve N, Soler C, Frieiro E, González JM (2014). Genetic diversity of SSR and ISSR markers in wild populations of *Brachypodium distachyon* and its close relatives *B. stacei* and *B. hybridum* (Poaceae). Plant Systematics and Evolution.

[ref-18] Hannah L, Flint L, Syphard AD, Moritz MA, Buckley LB, McCullough IM (2014). Fine-grain modeling of species’ response to climate change: holdouts, stepping-stones, and microrefugia. Trends in Ecology & Evolution.

[ref-19] Kamvar ZN, Tabima JF, Grünwald NJ (2014). *Poppr*: an R package for genetic analysis of populations with clonal, partially clonal, and/or sexual reproduction. PeerJ.

[ref-20] Korte MK, Manzaneda AJ, Martinez LM, Patterson TA, Etienne RS, van de Zande L, Smit C (2025). The effect of local perennial plants on the occurrence and traits of the *Brachypodium distachyon* complex along an aridity gradient. Plant Ecology.

[ref-21] Liancourt P, Tielbörger K (2011). Ecotypic differentiation determines the outcome of positive interactions in a dryland annual plant species. Perspectives in Plant Ecology, Evolution and Systematics.

[ref-22] Lortie CJ, Filazzola A, Westphal M, Butterfield HS (2022). Foundation plant species provide resilience and microclimatic heterogeneity in drylands. Scientific Reports.

[ref-23] Luzuriaga AL, Sánchez AM, Maestre FT, Escudero A (2012). Assemblage of a semi-arid annual plant community: abiotic and biotic filters act hierarchically. PLOS ONE.

[ref-24] Maestre FT, Bautista S, Cortina J, Bellot J (2001). Potential for using facilitation by grasses to establish shrubs on a semiarid degraded steppe. Ecological Applications.

[ref-25] Manzaneda AJ, Rey PJ, Anderson JT, Raskin E, Weiss-Lehman C, Mitchell-Olds T (2015). Natural variation, differentiation, and genetic trade-offs of ecophysiological traits in response to water limitation in *Brachypodium distachyon* and its descendent allotetraploid *B. hybridum* (Poaceae). Evolution.

[ref-26] Manzaneda AJ, Rey PJ, Bastida JM, Weiss-Lehman C, Raskin E, Mitchell-Olds T (2012). Environmental aridity is associated with cytotype segregation and polyploidy occurrence in *Brachypodium distachyon* (Poaceae). New Phytologist.

[ref-27] Manzano P, Malo JE (2006). Extreme long-distance seed dispersal via sheep. Frontiers in Ecology and the Environment.

[ref-28] Marques I, Shiposha V, López-Alvarez D, Manzaneda AJ, Hernandez P, Olonova M, Catalán P (2017). Environmental isolation explains Iberian genetic diversity in the highly homozygous model grass *Brachypodium distachyon*. BMC Evolutionary Biology.

[ref-29] Michalet R, Le Bagousse-Pinguet Y, Maalouf J-P, Lortie CJ (2014). Two alternatives to the stress-gradient hypothesis at the edge of life: the collapse of facilitation and the switch from facilitation to competition. Journal of Vegetation Science.

[ref-30] Morelli TL, Maher SP, Marisa Lim CW, Kastely C, Eastman LM, Flint LE, Flint AL, Beissinger SR, Moritz C (2017). Climate change refugia and habitat connectivity promote species persistence. Climate Change Responses.

[ref-31] Mu W, Li K, Yang Y, Breiman A, Yang J, Wu Y, Zhu M, Wang S, Catalan P, Nevo E (2023). Subgenomic stability of progenitor genomes during repeated allotetraploid origins of the same grass *Brachypodium hybridum*. Molecular Biology and Evolution.

[ref-32] Neji M, Geuna F, Taamalli W, Ibrahim Y, Chiozzotto R, Abdelly C, Gandour M (2015). Assessment of genetic diversity and population structure of Tunisian populations of *Brachypodium hybridum* by SSR markers. Flora - Morphology, Distribution, Functional Ecology of Plants.

[ref-33] O’Brien MJ, Carbonell EP, Losapio G, Schlüter PM, Schöb C (2021). Foundation species promote local adaptation and fine-scale distribution of herbaceous plants. Journal of Ecology.

[ref-34] Pritchard JK, Stephens M, Donnelly P (2000). Inference of population structure using multilocus genotype data. Genetics.

[ref-35] R Core Team (2024). R: A language and environment for statistical computing. https://www.r-project.org.

[ref-36] Richardson JL, Urban MC, Bolnick DI, Skelly DK (2014). Microgeographic adaptation and the spatial scale of evolution. Trends in Ecology & Evolution.

[ref-37] Ritland K (1990). Inferences about inbreeding depression based on changes of the inbreeding coefficient. Evolution.

[ref-38] Scarlett VT, Lovell JT, Shao M, Phillips J, Shu S, Lusinska J, Goodstein DM, Jenkins J, Grimwood J, Barry K, Chalhoub B, Schmutz J, Hasterok R, Catalán P, Vogel JP (2022). Multiple origins, one evolutionary trajectory: gradual evolution characterizes distinct lineages of allotetraploid Brachypodium. Genetics.

[ref-39] Schwartz CJ, Doyle MR, Manzaneda AJ, Rey PJ, Mitchell-Olds T, Amasino RM (2010). Natural variation of flowering time and vernalization responsiveness in *Brachypodium distachyon*. BioEnergy Research.

[ref-40] Shiposha V, Catalán P, Olonova M, Marques I (2016). Genetic structure and diversity of the selfing model grass *Brachypodium stacei* (Poaceae) in Western Mediterranean: out of the Iberian Peninsula and into the islands. PeerJ.

[ref-41] Shiposha V, Marques I, López-Alvarez D, Manzaneda AJ, Hernandez P, Olonova M, Catalán P (2020). Multiple founder events explain the genetic diversity and structure of the model allopolyploid grass *Brachypodium hybridum* in the Iberian Peninsula hotspot. Annals of Botany.

[ref-42] Tielbörger K, Kadmon R (1995). Effect of shrubs on emergence, survival and fecundity of four coexisting annual species in a sandy desert ecosystem. Écoscience.

[ref-43] Tilman D (1982). Resource competition and community structure.

[ref-44] Tirado R, Pugnaire FI (2003). Shrub spatial aggregation and consequences for reproductive success. Oecologia.

[ref-45] Tyler L, Fangel JU, Fagerström AD, Steinwand MA, Raab TK, Willats WGT, Vogel JP (2014). Selection and phenotypic characterization of a core collection of *Brachypodium distachyon* inbred lines. BMC Plant Biology.

[ref-46] Verdú M, Gómez JM, Valiente-Banuet A, Schöb C (2021). Facilitation and plant phenotypic evolution. Trends in Plant Science.

[ref-47] Vogel J, Tuna M, Budak H, Huo N, Gu YQ, Steinwand MA (2009). Development of SSR markers and analysis of diversity in Turkish populations of *Brachypodium distachyon*. BMC Plant Biology.

[ref-48] Weir BS, Cockerham CC (1984). Estimating F-statistics for the analysis of population structure. Evolution.

[ref-49] Woods DP, Ream TS, Bouché F, Lee J, Thrower N, Wilkerson C, Amasino RM (2017). Establishment of a vernalization requirement in *Brachypodium distachyon* requires repressor of vernalization1. Proceedings of the National Academy of Sciences of the United States of America.

[ref-50] Zomer RJ, Trabucco A, Bossio DA, Verchot LV (2008). Climate change mitigation: a spatial analysis of global land suitability for clean development mechanism afforestation and reforestation. Agriculture, Ecosystems & Environment.

[ref-51] Zomer RJ, Trabucco A, Van Straaten O, Bossio DA (2007). Carbon, land and water: a global analysis of the hydrologic dimensions of climate change mitigation through afforestation/reforestation.

