# Supplementary material for: The genetic structure of a Brachypodium hybridum population in a patchy arid landscape is independent of neighboring perennials and stable over two consecutive years
Source: PeerJ. 2026 Mar 2;14:e20787. doi: 10.7717/peerj.20787 (PMC12962130; doi:10.7717/peerj.20787)
Supplement: Supplemental Information 1 — Number of samples for each combination of canopy position (“in” = under perennial canopy; “out” = outside canopy) and sampling period. [file peerj-14-20787-s001.docx]

|  |  |  |  |
| --- | --- | --- | --- |
|  | Sampling period | Number of samples |  |
|  | In Fall 2018 | 11 |  |
|  | In Fall 2019 | 33 |  |
|  | In Spring 2018 | 40 |  |
|  | In Spring 2019 | 0 |  |
|  | Out Fall 2018 | 0 |  |
|  | Out Fall 2019 | 38 |  |
|  | Out Spring 2018 | 33 |  |
|  | Out Spring 2019 | 18 |  |
|  | Total | 173 |  |
