# Supplementary material for: The genetic structure of a Brachypodium hybridum population in a patchy arid landscape is independent of neighboring perennials and stable over two consecutive years
Source: PeerJ. 2026 Mar 2;14:e20787. doi: 10.7717/peerj.20787 (PMC12962130; doi:10.7717/peerj.20787)
Supplement: Supplemental Information 2 [file peerj-14-20787-s002.docx]

| Pop | Season | Year | ALB006d | ALB006s | ALB008s | ALB008d | ALB034d | AlB040d | ALB056s |
| --- | --- | --- | --- | --- | --- | --- | --- | --- | --- |
| In | Fall | 2018 | 343/343 | 374/382 | 102/102 | 116/116 | 221/221 | 183/183 | 237/237 |
| In | Fall | 2018 | 343/343 | 374/374 | 102/102 | 116/116 | 221/221 | 183/183 | 237/237 |
| In | Fall | 2018 | 343/343 | 374/374 | 102/102 | 116/116 | 221/221 | 183/183 | 237/237 |
| In | Fall | 2018 | 343/343 | 374/374 | 102/102 | 116/116 | 221/221 | 183/183 | 237/237 |
| In | Fall | 2018 | 343/343 | 374/374 | 102/102 | 116/116 | 235/235 | 186/186 | 235/235 |
| In | Fall | 2018 | 343/343 | 374/374 | 102/102 | 116/116 | 235/235 | 183/183 | 235/235 |
| In | Fall | 2018 | 343/343 | 374/374 | 102/102 | 116/116 | 235/235 | 186/186 | 235/235 |
| In | Fall | 2018 | 343/343 | 374/374 | 102/102 | 116/116 | 221/221 | 183/183 | 237/237 |
| In | Fall | 2018 |  |  | 102/102 | 116/116 | 221/221 | 186/186 | 237/237 |
| In | Fall | 2018 | 343/343 | 374/374 | 102/102 | 116/116 | 221/235 | 186/186 | 235/235 |
| In | Fall | 2018 | 343/343 | 380/380 | 102/102 | 116/116 | 221/221 | 186/186 | 235/235 |
| In | Fall | 2019 | 343/343 | 374/374 | 102/102 | 116/116 | 221/221 | 183/183 | 235/235 |
| In | Fall | 2019 | 343/343 | 374/374 | 102/102 | 116/116 | 235/235 | 183/183 | 235/235 |
| In | Fall | 2019 | 343/343 | 374/374 | 102/102 | 116/116 | 221/221 | 183/183 | 235/235 |
| In | Fall | 2019 | 343/343 | 374/374 | 102/102 | 116/116 | 235/235 | 186/186 | 235/235 |
| In | Fall | 2019 | 343/343 | 374/374 | 102/102 | 116/116 | 235/235 | 183/183 | 235/235 |
| In | Fall | 2019 | 343/343 | 374/374 | 102/102 | 116/116 | 235/235 | 186/186 | 235/235 |
| In | Fall | 2019 | 343/343 | 382/382 | 102/102 | 116/116 | 235/235 | 186/186 | 235/235 |
| In | Fall | 2019 | 343/343 | 374/374 | 102/102 | 116/116 | 235/235 | 186/186 | 235/235 |
| In | Fall | 2019 | 343/343 | 374/374 |  |  | 224/224 | 183/186 | 235/235 |
| In | Fall | 2019 | 343/343 | 374/374 | 102/102 | 116/116 | 235/235 | 186/186 | 235/235 |
| In | Fall | 2019 | 343/343 | 374/374 | 102/102 | 116/116 | 235/235 | 186/186 | 235/235 |
| In | Fall | 2019 | 343/343 | 374/374 | 102/102 | 116/116 | 235/235 | 186/186 | 235/235 |
| In | Fall | 2019 | 343/343 | 374/374 | 102/102 | 116/116 | 235/235 | 186/186 | 235/235 |
| In | Fall | 2019 | 343/343 | 374/374 | 102/102 | 116/116 | 221/221 | 183/183 | 237/237 |
| In | Fall | 2019 | 343/343 | 374/374 | 102/102 | 116/116 | 221/221 | 183/183 | 235/235 |
| In | Fall | 2019 | 343/343 | 374/374 | 102/102 | 116/116 | 235/235 | 186/186 |  |
| In | Fall | 2019 | 343/343 | 374/374 | 102/102 | 116/116 | 238/238 | 183/183 | 235/235 |
| In | Fall | 2019 | 343/343 | 359/359 | 102/102 | 116/116 | 227/227 | 183/183 | 251/251 |
| In | Fall | 2019 | 343/343 | 374/374 | 102/102 | 116/116 | 235/235 | 186/186 | 235/235 |
| In | Fall | 2019 | 343/343 | 374/374 | 102/102 | 116/116 | 235/235 | 186/186 | 235/235 |
| In | Fall | 2019 | 343/343 | 380/380 |  |  | 221/221 | 183/183 | 235/235 |
| In | Fall | 2019 | 343/343 | 380/380 | 102/102 | 116/116 | 235/235 | 183/183 | 235/235 |
| In | Fall | 2019 | 343/343 | 380/380 | 102/102 | 116/116 | 221/221 | 183/183 | 235/235 |
| In | Fall | 2019 | 343/343 | 374/374 | 102/102 | 116/116 | 235/235 | 186/186 | 235/235 |
| In | Fall | 2019 | 343/343 | 380/380 | 102/102 | 116/116 | 235/235 | 183/183 | 235/235 |
| In | Fall | 2019 | 343/343 | 374/374 | 102/102 | 116/116 | 235/235 | 186/186 | 235/235 |
| In | Fall | 2019 | 343/343 | 374/374 | 102/102 | 116/116 | 235/235 | 186/186 | 235/235 |
| In | Fall | 2019 | 343/343 | 374/374 | 102/102 | 116/116 | 235/235 | 186/186 | 235/235 |
| In | Fall | 2019 | 343/343 | 382/382 | 102/102 | 116/116 | 224/224 | 183/186 | 235/235 |
| In | Fall | 2019 | 343/343 | 374/374 | 102/102 | 116/116 | 235/235 | 186/186 | 235/235 |
| In | Fall | 2019 | 343/343 | 374/374 | 102/102 | 116/116 | 235/235 | 186/186 | 235/235 |
| In | Fall | 2019 | 343/343 | 374/374 |  |  | 235/235 | 186/186 | 235/235 |
| In | Fall | 2019 | 343/343 | 380/380 | 102/102 | 116/116 | 235/235 | 186/186 | 235/235 |
| Out | Fall | 2019 | 343/343 | 374/374 | 102/102 | 116/116 | 221/221 | 183/183 | 237/237 |
| Out | Fall | 2019 | 343/343 | 380/380 | 102/102 | 116/116 | 221/221 | 183/183 | 235/235 |
| Out | Fall | 2019 | 341/341 | 350/350 | 102/102 | 137/137 | 221/221 | 186/186 | 237/237 |
| Out | Fall | 2019 | 343/343 | 380/380 | 102/102 | 116/116 | 238/238 | 186/186 | 235/235 |
| Out | Fall | 2019 | 341/341 | 350/350 | 102/102 | 137/137 | 227/235 |  | 237/237 |
| Out | Fall | 2019 | 343/343 | 380/380 | 102/102 | 116/116 | 221/221 | 183/183 | 237/237 |
| Out | Fall | 2019 | 343/343 | 374/374 | 102/102 | 116/116 | 238/238 | 186/186 | 235/235 |
| Out | Fall | 2019 | 343/343 | 380/380 | 102/102 | 116/116 | 221/221 | 183/183 | 237/237 |
| Out | Fall | 2019 | 343/343 | 374/374 | 102/102 | 116/116 | 221/221 | 183/183 | 237/237 |
| Out | Fall | 2019 | 343/343 | 374/374 | 102/102 | 116/116 | 238/238 | 186/186 | 235/235 |
| Out | Fall | 2019 | 343/343 | 374/374 | 102/102 | 116/116 | 235/235 | 186/186 | 235/235 |
| Out | Fall | 2019 | 343/343 | 374/374 | 102/102 | 116/116 | 235/235 | 186/186 | 235/235 |
| Out | Fall | 2019 | 343/343 | 374/374 | 102/102 | 116/116 | 221/221 | 183/183 | 237/237 |
| Out | Fall | 2019 | 343/343 | 374/374 | 102/102 | 116/116 | 227/227 | 183/183 | 249/249 |
| Pop | Season | Year | ALB006d | ALB006s | ALB008s | ALB008d | ALB034d | AlB040d | ALB056s |
| Out | Fall | 2019 | 343/343 | 374/374 |  |  | 235/235 | 183/183 | 235/235 |
| Out | Fall | 2019 | 343/343 | 374/374 |  |  | 235/235 | 186/186 | 235/235 |
| Out | Fall | 2019 | 343/343 | 374/374 |  |  | 235/235 | 186/186 | 235/235 |
| Out | Fall | 2019 | 343/343 | 374/374 |  |  | 221/221 | 183/183 | 235/235 |
| Out | Fall | 2019 | 343/343 | 374/374 | 102/102 | 116/116 | 235/235 | 186/186 | 235/235 |
| Out | Fall | 2019 | 343/343 | 374/374 | 102/102 | 116/116 | 221/221 | 183/183 | 237/237 |
| Out | Fall | 2019 | 343/343 | 374/374 |  |  | 235/235 | 186/186 | 235/235 |
| Out | Fall | 2019 | 343/343 | 374/374 | 102/102 | 116/116 | 238/238 | 186/186 | 235/235 |
| Out | Fall | 2019 |  |  |  |  | 227/227 | 183/183 | 251/251 |
| Out | Fall | 2019 |  |  |  |  | 227/227 | 183/183 | 251/251 |
| Out | Fall | 2019 |  |  |  |  | 227/227 | 183/183 | 251/251 |
| Out | Fall | 2019 | 343/343 | 343/343 |  |  | 227/235 |  | 237/237 |
| Out | Fall | 2019 | 343/343 | 374/374 | 102/102 | 116/116 | 221/221 | 183/183 | 237/237 |
| Out | Fall | 2019 | 343/343 | 374/374 | 102/102 | 116/116 | 238/238 | 186/186 | 235/235 |
| Out | Fall | 2019 | 343/343 | 374/374 | 102/102 | 116/116 | 221/221 | 183/183 | 237/237 |
| Out | Fall | 2019 | 343/343 | 374/374 |  |  | 221/221 | 183/183 | 237/237 |
| Out | Fall | 2019 | 343/343 | 374/374 |  |  | 238/238 | 186/186 | 235/235 |
| Out | Fall | 2019 | 343/343 | 374/374 |  |  | 235/235 | 186/186 | 235/235 |
| Out | Fall | 2019 | 343/343 | 374/374 |  |  | 235/235 | 186/186 | 235/235 |
| Out | Fall | 2019 | 343/343 | 374/374 |  |  | 221/221 | 183/183 | 237/237 |
| Out | Fall | 2019 | 359/359 | 359/359 | 102/102 | 116/116 | 227/227 | 183/183 | 249/249 |
| Out | Fall | 2019 | 343/343 | 374/374 | 102/102 | 116/116 | 227/235 | 183/186 | 235/235 |
| Out | Fall | 2019 | 343/343 | 374/374 | 102/102 | 116/116 | 235/235 | 186/186 | 235/235 |
| In | Spring | 2018 | 343/343 | 374/374 | 102/102 | 116/116 | 235/235 | 186/186 | 235/235 |
| In | Spring | 2018 | 343/343 | 374/374 | 102/102 | 116/116 | 235/235 | 186/186 | 235/235 |
| In | Spring | 2018 | 343/343 | 374/374 | 102/102 | 116/116 | 221/221 | 183/183 | 237/237 |
| In | Spring | 2018 | 343/343 | 374/374 | 102/102 | 116/116 | 235/235 | 186/186 | 235/235 |
| In | Spring | 2018 | 343/343 | 380/380 | 102/102 | 116/116 | 235/235 | 183/183 | 235/235 |
| In | Spring | 2018 | 343/343 | 374/374 | 102/102 | 116/116 | 235/235 | 186/186 | 235/235 |
| In | Spring | 2018 | 343/343 | 380/380 |  |  | 235/235 | 183/183 | 235/235 |
| In | Spring | 2018 | 343/343 | 374/374 | 102/102 | 116/116 | 235/235 | 183/183 | 235/235 |
| In | Spring | 2018 | 343/343 | 380/380 | 102/102 | 116/116 | 221/221 | 183/183 | 235/235 |
| In | Spring | 2018 | 343/343 | 374/374 | 102/102 | 116/116 | 235/235 | 186/186 | 235/235 |
| In | Spring | 2018 | 343/343 | 374/374 | 102/102 | 116/116 | 221/221 | 183/183 | 237/237 |
| In | Spring | 2018 | 343/343 | 374/374 | 102/102 | 116/116 | 221/221 | 183/183 | 237/237 |
| In | Spring | 2018 | 343/343 | 374/374 | 102/102 | 116/116 | 235/235 | 186/186 | 235/235 |
| In | Spring | 2018 | 343/343 | 374/374 | 102/102 | 116/116 | 235/235 |  | 235/235 |
| In | Spring | 2018 | 343/343 | 374/374 | 102/102 | 116/116 | 221/221 | 183/183 | 237/237 |
| In | Spring | 2018 | 343/343 | 380/380 | 102/102 | 116/116 | 235/235 | 183/183 | 235/235 |
| In | Spring | 2018 | 343/343 | 380/380 | 102/102 | 116/116 | 221/221 | 183/183 | 235/235 |
| In | Spring | 2018 | 343/343 | 380/380 | 102/102 | 116/116 | 221/235 | 186/186 | 235/235 |
| In | Spring | 2018 | 343/343 | 374/374 | 102/102 | 116/116 | 235/235 | 186/186 | 235/235 |
| In | Spring | 2018 | 343/343 | 374/374 | 102/102 | 116/116 | 221/221 | 183/183 | 237/237 |
| In | Spring | 2018 | 343/343 | 374/374 | 102/102 | 116/116 | 235/235 | 186/186 | 235/235 |
| In | Spring | 2018 | 343/343 | 374/374 | 102/102 | 116/116 | 221/221 | 183/183 | 237/237 |
| In | Spring | 2018 | 343/343 | 374/374 | 102/102 | 116/116 | 235/235 | 186/186 | 235/235 |
| In | Spring | 2018 | 343/343 | 374/374 | 102/102 | 116/116 | 235/235 | 186/186 | 235/235 |
| In | Spring | 2018 | 343/343 | 374/374 | 102/102 | 116/116 | 235/235 | 186/186 | 235/235 |
| In | Spring | 2018 | 343/343 | 374/374 | 102/102 | 116/116 | 235/235 | 186/186 | 235/235 |
| In | Spring | 2018 | 343/343 | 374/374 | 102/102 | 116/116 | 235/235 | 186/186 | 235/235 |
| In | Spring | 2018 | 343/343 | 374/374 | 102/102 | 116/116 | 235/235 | 186/186 | 235/235 |
| In | Spring | 2018 | 343/343 | 380/380 | 102/102 | 116/116 |  | 186/186 | 235/235 |
| In | Spring | 2018 | 343/343 | 374/374 | 102/102 | 116/116 | 235/235 | 186/186 | 235/235 |
| In | Spring | 2018 | 343/343 | 374/374 | 102/102 | 116/116 | 235/235 | 186/186 | 235/235 |
| In | Spring | 2018 | 343/343 | 374/374 | 102/102 | 116/116 | 235/235 |  | 235/235 |
| In | Spring | 2018 | 343/343 | 374/374 | 102/102 | 116/116 | 235/235 | 183/183 | 235/235 |
| In | Summer | 2018 | 343/343 | 374/374 | 102/102 | 116/116 | 221/221 | 183/183 | 237/237 |
| In | Summer | 2018 | 343/343 | 374/374 | 102/102 | 116/116 | 221/221 | 183/183 | 235/235 |
| Pop | Season | Year | ALB006d | ALB006s | ALB008s | ALB008d | ALB034d | AlB040d | ALB056s |
| In | Summer | 2018 | 343/343 | 343/343 | 102/102 | 116/116 | 227/227 | 183/183 | 251/251 |
| In | Summer | 2018 | 343/343 | 382/382 | 102/102 | 116/116 | 235/235 | 186/186 | 235/235 |
| In | Summer | 2018 | 343/343 | 374/374 | 102/102 | 116/116 | 235/235 | 186/186 | 235/235 |
| Out | Spring | 2018 | 343/343 | 374/374 | 102/102 | 116/116 | 235/235 | 186/186 | 237/237 |
| Out | Spring | 2018 | 343/343 | 374/374 | 102/102 | 116/116 | 235/235 | 186/186 | 235/235 |
| Out | Spring | 2018 | 343/343 | 343/343 | 102/102 | 116/116 | 235/235 | 186/186 | 235/235 |
| Out | Spring | 2018 | 343/343 | 374/374 | 102/102 | 116/116 |  | 186/186 | 235/235 |
| Out | Spring | 2018 | 343/343 | 374/374 |  |  | 235/235 | 186/186 | 235/235 |
| Out | Spring | 2018 | 343/343 | 374/374 | 102/102 | 116/116 |  | 186/186 | 235/235 |
| Out | Spring | 2018 | 343/343 | 374/374 | 102/102 | 116/116 | 235/235 | 186/186 | 235/235 |
| Out | Spring | 2018 | 343/343 | 374/374 | 102/102 | 116/116 | 221/221 | 183/183 | 235/235 |
| Out | Spring | 2018 | 343/343 | 374/374 | 102/102 | 116/116 | 235/235 | 186/186 | 235/235 |
| Out | Spring | 2018 | 343/343 | 374/374 | 102/102 | 116/116 | 235/235 | 186/186 | 235/235 |
| Out | Spring | 2018 | 343/343 | 374/374 | 102/102 | 116/116 | 235/235 | 186/186 | 235/235 |
| Out | Spring | 2018 | 343/343 | 374/374 | 102/102 | 116/116 | 221/221 | 183/183 | 235/235 |
| Out | Spring | 2018 | 343/343 | 380/380 | 102/102 | 116/116 | 235/235 | 186/186 | 235/235 |
| Out | Spring | 2018 | 343/343 | 374/374 | 102/102 | 116/116 | 235/235 | 186/186 | 235/235 |
| Out | Spring | 2018 | 343/343 | 374/374 | 102/102 | 116/116 | 235/235 | 186/186 | 235/235 |
| Out | Spring | 2018 | 343/343 | 374/374 | 102/102 | 116/116 | 235/235 | 186/186 | 235/235 |
| Out | Summer | 2018 | 343/343 | 374/374 | 102/102 | 116/116 | 221/221 | 183/183 | 237/237 |
| Out | Summer | 2018 | 343/343 | 382/382 | 102/102 | 116/116 | 221/221 | 183/183 | 235/235 |
| Out | Summer | 2018 | 343/343 | 374/374 | 102/102 | 116/116 | 235/235 | 186/186 | / |
| Out | Summer | 2018 | 343/343 | 382/382 | 102/102 | 116/116 | 235/235 | 186/186 | 235/235 |
| Out | Summer | 2018 | 343/343 | 374/374 |  | 116/116 | 235/235 | 186/186 | 235/235 |
| Out | Summer | 2018 | 343/343 | 374/374 | 102/102 | 116/116 | 235/235 | 186/186 | 235/235 |
| Out | Spring | 2018 | 343/343 | 374/374 | 102/102 | 116/116 | 235/235 | 186/186 | 237/237 |
| Out | Spring | 2018 | 343/343 | 374/374 | 102/102 | 116/116 | 221/221 | 183/183 | 235/235 |
| Out | Spring | 2018 | 343/343 | 374/374 | 102/102 | 116/116 | 235/235 | 186/186 | 237/237 |
| Out | Spring | 2018 | 343/343 | 374/374 | 102/102 | 116/116 | 221/221 | 183/183 | 235/235 |
| Out | Spring | 2018 | 343/343 | 374/374 | 102/102 | 116/116 | 235/235 | 186/186 | 235/235 |
| Out | Summer | 2018 | 343/343 | 374/374 | 102/102 | 116/116 | 221/221 | 183/183 | 237/237 |
| Out | Summer | 2018 | 343/343 | 374/374 | 102/102 | 116/116 | 235/235 | 186/186 | 235/235 |
| Out | Summer | 2018 | 343/343 | 374/374 | 102/102 | 116/116 | 235/235 | 186/186 | 235/235 |
| Out | Spring | 2018 | 343/343 | 374/374 | 102/102 | 116/116 | 235/235 | 186/186 | 235/235 |
| Out | Spring | 2018 | 343/343 | 374/374 | 102/102 | 116/116 | 221/221 | 183/183 | 237/237 |
| Out | Spring | 2018 | 343/343 | 374/374 | 102/102 | 116/116 | 235/235 | 186/186 | 235/235 |
| Out | Spring | 2019 | 343/343 | 374/374 | 102/102 | 116/116 | 221/221 | 183/183 | 235/235 |
| Out | Spring | 2019 | 343/343 | 374/374 | 102/102 | 116/116 | 235/235 | 186/186 | 235/235 |
| Out | Spring | 2019 | 343/343 | 374/374 | 102/102 | 116/116 | 235/235 | 183/183 | 237/237 |
| Out | Spring | 2019 | 343/343 | 374/374 | 102/102 | 116/116 | 221/221 | 183/183 | 237/237 |
| Out | Spring | 2019 | 343/343 | 374/374 | 102/102 | 116/116 | 235/235 | 186/186 | 235/235 |
| Out | Spring | 2019 | 343/343 | 374/374 | 102/102 | 116/116 |  |  | 235/235 |
| Out | Spring | 2019 | 343/343 | 374/374 | 102/102 | 116/116 | 221/221 | 183/183 | 235/235 |
| Out | Spring | 2019 | 343/343 | 374/374 | 102/102 | 116/116 | 235/235 | 186/186 | 235/235 |
| Out | Spring | 2019 | 343/343 | 374/374 | 102/102 | 116/116 | 235/235 | 186/186 | 235/235 |
| Out | Spring | 2019 | 343/343 | 374/374 | 102/102 | 116/116 | 235/235 | 186/186 | 235/235 |
| Out | Spring | 2019 | 343/343 | 374/374 | 102/102 | 116/116 | 235/235 | 186/186 | 235/235 |
| Out | Spring | 2019 | 343/343 | 374/374 | 102/102 | 116/116 | 235/235 | 186/186 | 237/237 |
| Out | Spring | 2019 | 343/343 | 374/374 | 102/102 | 116/116 | 221/221 | 183/183 | 235/235 |
| Out | Spring | 2019 | 343/343 | 382/382 | 102/102 | 116/116 | 218/218 | 171/171 | 235/235 |
| Out | Spring | 2019 | 343/343 | 374/374 | 102/102 | 116/116 | 235/235 | 183/183 | 237/237 |
| Out | Spring | 2019 | 343/343 | 380/380 | 102/102 | 116/116 | 221/221 | 183/183 | 235/235 |
| Out | Spring | 2019 | 343/343 | 374/374 | 102/102 | 116/116 | 238/238 | 186/186 | 235/235 |
| Out | Spring | 2019 | 343/343 | 380/380 | 102/102 | 116/116 | 235/235 | 183/183 | 235/235 |

| Pop | Season | Year | ALB056d | ALB086d | ALB087d | ALB089d | ALB100s | ALB100d | ALB131d |
| --- | --- | --- | --- | --- | --- | --- | --- | --- | --- |
| In | Fall | 2018 | 251/251 | 191/191 | 194/194 | 201/201 | 238/238 | 244/244 | 286/286 |
| In | Fall | 2018 | 251/251 | 191/191 | 194/194 | 201/201 | 238/238 | 244/244 | 286/286 |
| In | Fall | 2018 | 251/251 | 191/191 | 194/194 | 201/201 | 238/238 | 244/244 | 286/286 |
| In | Fall | 2018 | 251/251 | 191/191 | 194/194 | 201/201 | 238/238 | 244/244 | 284/284 |
| In | Fall | 2018 | 251/251 | 191/191 | 203/203 | 201/201 | 238/238 | 272/272 | 286/286 |
| In | Fall | 2018 | 251/251 | 191/191 | 194/194 | 199/199 |  |  | 286/286 |
| In | Fall | 2018 | 251/251 | 191/191 | 203/203 | 199/199 | 238/238 | 244/244 | 286/286 |
| In | Fall | 2018 | 251/251 | 191/191 | 194/194 | 201/201 | 238/238 | 244/244 | 286/286 |
| In | Fall | 2018 | 251/251 | 191/191 | 194/194 | 199/199 | 238/238 | 244/244 | 286/286 |
| In | Fall | 2018 | 251/251 | 191/191 | 194/203 | 199/199 | 238/238 | 244/272 | 286/286 |
| In | Fall | 2018 | 249/249 | 191/191 | 194/194 | 199/199 | 238/238 | 272/272 | 286/286 |
| In | Fall | 2019 | 249/249 | 191/191 | 196/196 | 201/201 | 238/238 | 244/244 | 286/286 |
| In | Fall | 2019 | 251/251 | 191/191 | 194/194 | 201/201 | 238/238 | 244/244 | 286/286 |
| In | Fall | 2019 | 249/249 | 191/191 | 196/196 | 199/199 | 238/238 | 244/244 | 286/286 |
| In | Fall | 2019 | 251/251 | 191/191 | 203/203 | 199/199 | 238/238 | 244/244 | 286/286 |
| In | Fall | 2019 | 251/251 | 191/191 | 194/194 | 201/201 | 244/244 | 244/244 | 286/286 |
| In | Fall | 2019 | 251/251 | 191/191 | 203/203 | 199/199 | 238/238 | 244/244 | 286/286 |
| In | Fall | 2019 | 251/251 | 191/191 | 203/203 | 201/201 | 238/238 | 244/244 | 286/286 |
| In | Fall | 2019 | 251/251 | 191/191 | 203/203 | 199/199 | 238/238 | 244/244 | 286/286 |
| In | Fall | 2019 | 251/251 | 191/191 | 194/194 | 199/199 | 238/238 | 244/244 | 286/286 |
| In | Fall | 2019 | 251/251 | 191/191 | 203/203 | 199/199 | 238/238 | 244/244 | 286/286 |
| In | Fall | 2019 | 251/251 | 191/191 | 203/203 | 201/201 | 238/238 | 244/244 | 286/286 |
| In | Fall | 2019 | 251/251 | 191/191 | 203/203 | 199/199 | 238/238 | 244/244 | 286/286 |
| In | Fall | 2019 | 251/251 | 191/191 | 203/203 | 199/199 | 238/238 | 244/244 | 286/286 |
| In | Fall | 2019 | 251/251 | 191/191 | 194/194 | 201/201 | 238/238 | 244/244 | 286/286 |
| In | Fall | 2019 | 251/251 | 191/191 | 203/203 | 199/199 | 238/238 | 244/244 | 286/286 |
| In | Fall | 2019 |  | 191/191 |  | 199/199 | 238/238 | 244/244 |  |
| In | Fall | 2019 | 251/251 | 191/191 | 196/196 | 199/199 | 238/238 | 244/244 | 286/286 |
| In | Fall | 2019 | 251/251 | 191/191 |  | 199/199 |  |  |  |
| In | Fall | 2019 | 251/251 | 191/191 | 203/203 | 199/199 | 238/238 | 244/244 | 286/286 |
| In | Fall | 2019 | 251/251 | 191/191 | 203/203 | 199/199 | 238/238 | 244/244 | 286/286 |
| In | Fall | 2019 | 249/249 | 191/191 | 196/196 | 201/201 |  |  | 286/286 |
| In | Fall | 2019 | 251/251 | 191/191 | 194/194 | 199/199 | 238/238 | 244/244 | 286/286 |
| In | Fall | 2019 | 249/249 | 191/191 | 196/196 | 199/199 | 238/238 | 258/258 | 286/286 |
| In | Fall | 2019 | 251/251 | 191/191 | 203/203 | 199/199 | 238/238 | 238/238 | 286/286 |
| In | Fall | 2019 | 251/251 | 191/191 | 194/194 | 199/199 | 238/238 | 244/244 | 286/286 |
| In | Fall | 2019 | 251/251 | 191/191 | 203/203 | 199/199 |  |  | 286/286 |
| In | Fall | 2019 | 251/251 | 191/191 | 203/203 | 199/199 | 238/238 | 238/238 | 286/286 |
| In | Fall | 2019 | 251/251 | 191/191 | 203/203 | 199/199 |  |  | 286/286 |
| In | Fall | 2019 | 251/251 | 191/191 | 194/194 | 201/201 | 238/238 | 238/238 | 286/286 |
| In | Fall | 2019 | 251/251 | 191/191 | 203/203 | 199/199 | 238/238 | 244/244 | 286/286 |
| In | Fall | 2019 | 251/251 | 191/191 | 203/203 | 199/199 | 238/238 | 244/244 | 286/286 |
| In | Fall | 2019 | 251/251 | 191/191 | 203/203 | 199/199 | 238/238 | 244/244 | 286/286 |
| In | Fall | 2019 | 251/251 | 191/191 | 203/203 | 199/199 |  |  | 286/286 |
| Out | Fall | 2019 | 251/251 | 191/191 | 194/194 | 201/201 | 238/238 | 244/244 | 286/286 |
| Out | Fall | 2019 | 251/251 | 191/191 | 194/194 | 199/199 | 238/238 | 244/244 | 286/286 |
| Out | Fall | 2019 |  |  | 196/196 | 199/199 | 238/238 | 244/244 | 292/292 |
| Out | Fall | 2019 | 251/251 | 191/191 | 203/203 | 201/201 | 238/238 | 258/258 | 286/286 |
| Out | Fall | 2019 | 251/251 | 205/205 | 196/203 | 199/199 | 244/244 | 244/244 | 286/286 |
| Out | Fall | 2019 | 251/251 | 191/191 | 194/194 | 201/201 | 238/238 | 272/272 | 286/286 |
| Out | Fall | 2019 | 251/251 | 191/191 | 203/203 | 199/199 | 238/238 | 244/244 | 286/286 |
| Out | Fall | 2019 | 251/251 | 191/191 | 194/194 | 201/201 | 238/238 | 272/272 | 286/286 |
| Out | Fall | 2019 | 251/251 | 191/191 | 194/194 | 199/199 | 238/238 | 244/244 | 286/286 |
| Out | Fall | 2019 | 251/251 | 191/191 | 203/203 | 201/201 | 238/238 | 244/244 | 286/286 |
| Out | Fall | 2019 | 251/251 | 191/191 | 203/203 | 199/199 | 238/238 | 244/244 | 286/286 |
| Out | Fall | 2019 | 251/251 | 191/191 | 203/203 | 199/199 | 238/238 | 244/244 | 286/286 |
| Out | Fall | 2019 | 251/251 | 191/191 | 194/194 | 201/201 | 238/238 | 244/244 | 286/286 |
| Out | Fall | 2019 | 249/249 | 191/191 | 203/203 | 201/201 | 238/238 | 244/244 | 286/286 |
| Pop | Season | Year | ALB056d | ALB086d | ALB087d | ALB089d | ALB100s | ALB100d | ALB131d |
| Out | Fall | 2019 | 251/251 | 191/191 | 194/194 | 199/199 | 238/238 | 244/244 | 286/286 |
| Out | Fall | 2019 | 251/251 | 191/191 | 203/203 | 199/199 | 238/238 | 244/244 | 286/286 |
| Out | Fall | 2019 | 251/251 | 191/191 | 203/203 | 199/199 | 238/238 | 244/244 | 286/286 |
| Out | Fall | 2019 | 251/251 | 191/191 |  | 199/199 | 238/238 | 244/244 | 286/286 |
| Out | Fall | 2019 | 251/251 | 191/191 | 203/203 | 199/199 | 238/238 | 244/244 | 286/286 |
| Out | Fall | 2019 | 251/251 | 191/191 | 194/194 | 201/201 | 238/238 | 244/244 | 286/286 |
| Out | Fall | 2019 | 251/251 | 191/191 | 194/194 | 199/199 | 238/238 | 244/244 | 286/286 |
| Out | Fall | 2019 | 251/251 | 191/191 | 203/203 |  | 238/238 | 272/272 | 286/286 |
| Out | Fall | 2019 | 251/251 | 191/191 | 194/194 | 206/206 |  |  | 286/286 |
| Out | Fall | 2019 | 251/251 | 191/191 | 194/194 | 206/206 |  |  | 286/286 |
| Out | Fall | 2019 | 251/251 | 191/191 | 194/194 | 206/206 |  |  | 286/286 |
| Out | Fall | 2019 | 251/251 | 191/191 | 196/203 | 199/199 | 238/238 | 244/244 | 286/286 |
| Out | Fall | 2019 | 251/251 | 191/191 | 194/194 | 201/201 | 238/238 | 244/244 | 286/286 |
| Out | Fall | 2019 | 251/251 | 191/191 | 203/203 | 201/201 | 238/238 | 238/238 | 286/286 |
| Out | Fall | 2019 | 251/251 | 191/191 | 194/194 | 201/201 | 238/238 | 244/244 | 286/286 |
| Out | Fall | 2019 | 251/251 | 191/191 | 194/194 | 201/201 | 238/238 | 244/244 | 286/286 |
| Out | Fall | 2019 | 251/251 | 191/191 | 203/203 | 201/201 | 238/238 | 238/238 | 286/286 |
| Out | Fall | 2019 | 251/251 | 191/191 | 203/203 | 199/199 |  |  | 286/286 |
| Out | Fall | 2019 | 251/251 | 191/191 | 203/203 | 199/199 | 238/238 | 244/244 | 286/286 |
| Out | Fall | 2019 | 251/251 | 191/191 | 194/194 | 201/201 | 238/238 | 244/244 | 286/286 |
| Out | Fall | 2019 | 249/249 | 197/197 | 203/203 | 206/206 |  |  | 286/286 |
| Out | Fall | 2019 | 251/251 | 191/191 | 196/203 | 199/199 | 238/238 | 244/244 | 286/286 |
| Out | Fall | 2019 | 251/251 | 191/191 | 203/203 | 199/199 | 238/238 | 238/238 |  |
| In | Spring | 2018 | 251/251 | 191/191 | 203/203 | 199/199 | 238/238 | 244/244 | 286/286 |
| In | Spring | 2018 | 251/251 | 191/191 | 203/203 | 199/199 | 238/238 | 244/244 | 286/286 |
| In | Spring | 2018 | 251/251 | 191/191 | 194/194 | 201/201 |  |  | 286/286 |
| In | Spring | 2018 | 251/251 | 191/191 | 203/203 | 199/199 | 238/238 | 244/244 | 286/286 |
| In | Spring | 2018 | 251/251 | 191/191 | 194/194 | 199/199 | 238/238 | 244/244 | 286/286 |
| In | Spring | 2018 | 251/251 | 191/191 | 203/203 | 199/199 | 238/238 | 244/244 | 286/286 |
| In | Spring | 2018 | 251/251 | 191/191 | 194/194 | 199/199 | 238/238 | 244/244 | 286/286 |
| In | Spring | 2018 | 251/251 | 191/191 | 194/194 | 199/199 | 238/238 | 272/272 | 286/286 |
| In | Spring | 2018 | 249/249 | 191/191 | 196/196 | 201/201 | 238/238 | 258/258 | 286/286 |
| In | Spring | 2018 | 251/251 | 191/191 | 203/203 | 199/199 | 238/238 | 244/244 | 286/286 |
| In | Spring | 2018 | 251/251 | 191/191 | 194/194 | 201/201 | 238/238 | 244/244 | 286/286 |
| In | Spring | 2018 | 251/251 | 191/191 | 194/194 | 201/201 | 238/238 | 244/244 | 286/286 |
| In | Spring | 2018 | 251/251 | 191/191 | 203/203 | 199/199 | 238/238 | 244/244 | 286/286 |
| In | Spring | 2018 | 251/251 | 191/191 | 203/203 | 199/199 | 238/238 | 244/244 | 286/286 |
| In | Spring | 2018 | 251/251 | 191/191 | 194/194 | 201/201 |  |  | 286/286 |
| In | Spring | 2018 | 251/251 | 191/191 | 194/194 | 199/199 | 238/238 | 244/244 | 286/286 |
| In | Spring | 2018 | 249/249 | 191/191 | 196/196 | 201/201 | 238/238 | 258/258 | 286/286 |
| In | Spring | 2018 | 251/251 | 191/191 | 194/196 | 199/199 | 238/238 | 244/258 | 286/286 |
| In | Spring | 2018 | 251/251 | 191/191 | 203/203 | 199/199 | 238/238 | 244/244 | 286/286 |
| In | Spring | 2018 | 251/251 | 191/191 | 194/194 | 201/201 | 238/238 | 244/244 | 286/286 |
| In | Spring | 2018 | 251/251 | 191/191 | 203/203 | 199/199 | 238/238 | 244/244 | 286/286 |
| In | Spring | 2018 | 251/251 | 191/191 | 194/194 | 201/201 | 238/238 | 244/244 | 286/286 |
| In | Spring | 2018 | 251/251 | 191/191 | 203/203 | 199/199 | 238/238 | 244/244 | 286/286 |
| In | Spring | 2018 | 251/251 | 191/191 | 203/203 | 199/199 | 238/238 | 244/244 | 286/286 |
| In | Spring | 2018 | 251/251 | 191/191 | 203/203 | 199/199 | 238/238 | 244/244 | 286/286 |
| In | Spring | 2018 | 251/251 | 191/191 | 203/203 | 199/199 | 238/238 | 244/244 | 286/286 |
| In | Spring | 2018 | 251/251 | 191/191 | 203/203 | 199/199 | 238/238 | 244/244 | 286/286 |
| In | Spring | 2018 | 251/251 | 191/191 | 203/203 | 199/199 | 238/238 | 244/244 | 286/286 |
| In | Spring | 2018 | 251/251 | 191/191 | 194/194 | 199/199 | 238/238 | 238/238 | 286/286 |
| In | Spring | 2018 | 251/251 | 191/191 | 203/203 | 199/199 | 238/238 | 244/244 | 286/286 |
| In | Spring | 2018 | 251/251 | 191/191 | 203/203 | 199/199 | 238/238 | 244/244 | 286/286 |
| In | Spring | 2018 | 251/251 | 191/191 | 203/203 | 199/199 | 238/238 | 244/244 | 286/286 |
| In | Spring | 2018 | 251/251 | 191/191 | 194/194 | 199/199 | 238/238 | 244/244 | 286/286 |
| In | Summer | 2018 | 251/251 | 191/191 | 194/194 | 199/199 | 238/238 | 244/244 | 286/286 |
| In | Summer | 2018 | 251/251 | 191/191 | 203/203 | 201/201 | 238/238 | 244/244 | 286/286 |
| In | Summer | 2018 |  | 191/191 |  | 199/199 | 238/238 | 244/244 |  |
| Pop | Season | Year | ALB056d | ALB086d | ALB087d | ALB089d | ALB100s | ALB100d | ALB131d |
| In | Summer | 2018 | 251/251 | 191/191 |  | 199/199 | 238/238 | 244/244 |  |
| In | Summer | 2018 | 251/251 | 191/191 | 203/203 | 201/201 | 238/238 | 244/244 | 286/286 |
| In | Summer | 2018 | 251/251 | 191/191 | 203/203 | 199/199 | 238/238 | 244/244 | 286/286 |
| Out | Spring | 2018 | 251/251 | 191/191 | 194/194 | 201/201 | 238/238 | 244/244 | 286/286 |
| Out | Spring | 2018 | 251/251 | 191/191 | 203/203 | 199/199 | 238/238 | 244/244 | 286/286 |
| Out | Spring | 2018 | 251/251 |  |  |  | 238/238 | 244/244 | 286/286 |
| Out | Spring | 2018 | 251/251 | 191/191 | 203/203 | 199/199 | 238/238 | 238/238 | 286/286 |
| Out | Spring | 2018 | 251/251 | 191/191 |  |  | 238/238 | 244/244 | 286/286 |
| Out | Spring | 2018 | 251/251 |  | 203/203 | 199/199 | 238/238 | 244/244 | 286/286 |
| Out | Spring | 2018 | 251/251 |  | 203/203 | 199/199 | 238/238 | 244/244 | 286/286 |
| Out | Spring | 2018 | 251/251 | 191/191 | 203/203 | 199/199 | 238/238 | 244/244 | 286/286 |
| Out | Spring | 2018 | 251/251 | 191/191 | 203/203 | 199/199 | 238/238 | 244/244 | 286/286 |
| Out | Spring | 2018 | 251/251 | 191/191 | 203/203 | 199/199 | 238/238 | 244/244 | 286/286 |
| Out | Spring | 2018 | 251/251 | 191/191 | 203/203 | 199/199 | 238/238 | 244/244 | 286/286 |
| Out | Spring | 2018 | 251/251 | 191/191 | 203/203 | 199/199 | 238/238 | 244/244 | 286/286 |
| Out | Spring | 2018 | 249/249 | 191/191 | 196/196 | 201/201 | 238/238 | 258/258 | 286/286 |
| Out | Spring | 2018 | 251/251 | 191/191 | 203/203 | 199/199 | 238/238 | 244/244 | 286/286 |
| Out | Spring | 2018 | 251/251 | 191/191 | 203/203 | 199/199 | 238/238 | 244/244 | 286/286 |
| Out | Spring | 2018 | 251/251 | 191/191 | 203/203 | 199/199 | 238/238 | 244/244 | 286/286 |
| Out | Summer | 2018 | 251/251 | 191/191 | 194/194 | 201/201 | 238/238 | 244/244 | 286/286 |
| Out | Summer | 2018 | 249/249 | 191/191 | 194/194 | 199/199 | 238/238 | 244/244 | 286/286 |
| Out | Summer | 2018 | 251/251 | 191/191 | 203/203 | 199/199 | 238/238 | 244/244 | 286/286 |
| Out | Summer | 2018 | 249/249 | 191/191 | 194/194 | 201/201 | 238/238 | 244/244 | 286/286 |
| Out | Summer | 2018 | 251/251 | 191/191 | 203/203 | 199/199 | 238/238 | 244/244 | 286/286 |
| Out | Summer | 2018 | 251/251 | 191/191 | 203/203 |  | 238/238 | 244/244 | 286/286 |
| Out | Spring | 2018 | 251/251 | 191/191 | 194/194 | 201/201 | 238/238 | 244/244 | 286/286 |
| Out | Spring | 2018 | 251/251 | 191/191 | 203/203 | 199/199 | 238/238 | 238/238 | 286/286 |
| Out | Spring | 2018 | 251/251 | 191/191 | 194/194 | 201/201 | 238/238 | 244/244 | 286/286 |
| Out | Spring | 2018 | 251/251 | 191/191 | 194/194 | 199/199 | 238/238 | 244/244 | 286/286 |
| Out | Spring | 2018 | 251/251 | 191/191 | 203/203 | 199/199 | 238/238 | 244/244 | 286/286 |
| Out | Summer | 2018 | 251/251 | 191/191 | 194/194 | 199/199 | 238/238 | 244/244 | 286/286 |
| Out | Summer | 2018 | 251/251 | 191/191 | 203/203 | 201/201 | 238/238 | 272/272 | 286/286 |
| Out | Summer | 2018 | 251/251 | 191/191 | 203/203 | 199/199 | 238/238 | 244/244 | 286/286 |
| Out | Spring | 2018 | 251/251 | 191/191 | 203/203 | 199/199 | 238/238 | 244/244 | 286/286 |
| Out | Spring | 2018 | 251/251 | 191/191 | 194/194 | 201/201 | 238/238 | 244/244 | 286/286 |
| Out | Spring | 2018 | 251/251 | 191/191 | 194/194 | 201/201 | 238/238 | 244/244 | 286/286 |
| Out | Spring | 2019 | 251/251 | 191/191 | 203/203 | 199/199 | 238/238 | 244/244 | 286/286 |
| Out | Spring | 2019 | 251/251 | 191/191 | 203/203 | 199/199 | 238/238 | 244/244 | 286/286 |
| Out | Spring | 2019 | 251/251 | 191/191 | 194/194 | 201/201 | 238/238 | 244/244 | 286/286 |
| Out | Spring | 2019 | 251/251 | 191/191 | 194/194 | 201/201 | 238/238 | 244/244 | 286/286 |
| Out | Spring | 2019 | 251/251 | 191/191 | 203/203 | 199/199 | 238/238 | 244/244 | 286/286 |
| Out | Spring | 2019 | 249/249 | 191/191 | 196/196 | 199/199 | 238/238 | 244/258 | 286/286 |
| Out | Spring | 2019 | 251/251 | 191/191 | 203/203 | 199/199 | 238/238 | 244/244 | 286/286 |
| Out | Spring | 2019 | 251/251 | 191/191 | 203/203 | 199/199 | 238/238 | 244/244 | 286/286 |
| Out | Spring | 2019 | 251/251 | 191/191 | 203/203 | 199/199 | 238/238 | 238/238 | 286/286 |
| Out | Spring | 2019 | 251/251 | 191/191 | 203/203 | 199/199 | 238/238 | 244/244 | 286/286 |
| Out | Spring | 2019 | 251/251 | 191/191 | 203/203 | 199/199 | 238/238 | 244/244 | 286/286 |
| Out | Spring | 2019 | 251/251 | 191/191 | 194/194 | 201/201 | 238/238 | 244/244 | 286/286 |
| Out | Spring | 2019 | 251/251 | 191/191 | 203/203 | 199/199 | 238/238 | 244/244 | 286/286 |
| Out | Spring | 2019 | 251/251 | 191/191 | 194/194 | 199/199 | 238/238 | 272/272 | 286/286 |
| Out | Spring | 2019 | 251/251 | 191/191 | 194/194 | 201/201 | 238/238 | 244/244 | 286/286 |
| Out | Spring | 2019 | 249/249 | 191/191 | 196/196 | 201/201 | 238/238 | 258/258 | 286/286 |
| Out | Spring | 2019 | 251/251 | 191/191 | 194/194 | 199/199 | 238/238 | 238/238 | 286/286 |
| Out | Spring | 2019 | 251/251 | 191/191 | 194/194 | 201/201 | 238/238 | 272/272 | 286/286 |

| Pop | Season | Year | ALB139s | ALB139d | ALB158d | ALB165s | ALB165d | ALB230d | ALB256d |
| --- | --- | --- | --- | --- | --- | --- | --- | --- | --- |
| In | Fall | 2018 | 294/294 | 319/319 | 234/234 | 158/158 | 182/182 | 271/271 | 187/187 |
| In | Fall | 2018 | 294/294 | 319/319 | 234/234 | 158/158 | 182/182 | 271/271 | 187/187 |
| In | Fall | 2018 | 294/294 | 319/319 | 234/234 | 158/158 | 182/182 | 271/271 | 187/187 |
| In | Fall | 2018 | 294/294 | 319/319 | 234/234 | 158/158 | 191/191 | 271/271 | 187/187 |
| In | Fall | 2018 | 294/294 | 319/319 | 237/237 | 158/158 | 182/182 | 271/271 | 188/188 |
| In | Fall | 2018 | 294/294 | 319/319 | 234/234 | 158/158 | 191/191 | 271/271 | 187/187 |
| In | Fall | 2018 | 294/294 | 319/319 | 234/234 | 158/158 | 191/191 | 271/271 | 187/187 |
| In | Fall | 2018 | 294/294 | 319/319 | 234/234 | 158/158 | 182/182 | 271/271 | 187/187 |
| In | Fall | 2018 | 294/294 | 319/319 | 234/234 | 158/158 | 191/191 | 271/271 | 187/187 |
| In | Fall | 2018 | 294/294 | 319/319 | 234/234 | 158/158 | 191/191 | 271/271 | 187/187 |
| In | Fall | 2018 | 294/294 | 319/319 | 234/234 | 158/158 | 194/194 | 271/271 | 187/187 |
| In | Fall | 2019 | 294/294 | 319/319 | 234/234 | 158/158 | 182/182 | 271/271 | 187/187 |
| In | Fall | 2019 | 294/294 | 319/319 | 234/234 | 158/158 | 185/185 | 271/271 | 187/187 |
| In | Fall | 2019 | 294/294 | 319/319 | 234/234 | 158/158 | 182/182 | 271/271 | 187/187 |
| In | Fall | 2019 | 294/294 | 319/319 | 234/234 | 158/158 | 191/191 | 271/271 | 187/187 |
| In | Fall | 2019 | 294/294 | 319/319 | 234/234 | 158/158 | 191/191 | 271/271 | 187/187 |
| In | Fall | 2019 | 294/294 | 319/319 | 234/234 | 158/158 | 191/191 | 271/271 | 187/187 |
| In | Fall | 2019 | 294/294 | 319/319 | 234/234 | 158/158 | 191/191 | 271/271 | 187/187 |
| In | Fall | 2019 | 294/294 | 319/319 | 234/234 | 158/158 | 191/191 | 271/271 | 187/187 |
| In | Fall | 2019 | 294/294 | 319/319 | 234/234 |  |  | 271/271 | 188/188 |
| In | Fall | 2019 | 294/294 | 319/319 | 234/234 | 158/158 | 191/191 | 271/271 | 187/187 |
| In | Fall | 2019 | 294/294 | 319/319 | 234/234 |  |  | 271/271 | 187/187 |
| In | Fall | 2019 | 294/294 | 319/319 | 234/234 | 158/158 | 191/191 | 271/271 | 187/187 |
| In | Fall | 2019 | 294/294 | 319/319 | 234/234 |  |  | 271/271 | 187/187 |
| In | Fall | 2019 |  |  | 234/234 | 158/158 | 182/182 | 271/271 | 187/187 |
| In | Fall | 2019 |  |  | 234/234 | 158/158 | 191/191 | 271/271 | 187/187 |
| In | Fall | 2019 |  |  | 234/234 | 158/158 | 191/191 | 271/271 | 187/187 |
| In | Fall | 2019 |  |  | 234/234 | 158/158 | 182/182 | 271/271 | 188/188 |
| In | Fall | 2019 |  |  | 234/234 | 158/158 | 158/158 | 271/271 | 187/187 |
| In | Fall | 2019 |  |  | 234/234 | 158/158 | 191/191 | 271/271 | 187/187 |
| In | Fall | 2019 |  |  | 234/234 | 158/158 | 191/191 | 271/271 | 187/187 |
| In | Fall | 2019 |  |  | 234/234 | 158/158 | 182/182 | 271/271 | 187/187 |
| In | Fall | 2019 |  |  | 237/237 | 158/158 | 185/185 | 271/271 | 187/187 |
| In | Fall | 2019 |  |  | 234/234 | 158/158 | 182/182 | 271/271 | 187/187 |
| In | Fall | 2019 |  |  | 234/234 | 158/158 | 191/191 | 271/271 | 187/187 |
| In | Fall | 2019 |  |  | 237/237 | 158/158 | 191/191 | 271/271 | 187/187 |
| In | Fall | 2019 |  |  | 234/234 | 158/158 | 191/191 | 271/271 | 187/187 |
| In | Fall | 2019 |  |  | 234/234 | 158/158 | 191/191 | 271/271 | 187/187 |
| In | Fall | 2019 |  |  | 234/234 | 158/158 | 191/191 | 271/271 | 187/187 |
| In | Fall | 2019 |  |  | 234/234 |  |  | 271/271 | 188/188 |
| In | Fall | 2019 |  |  | 234/234 | 158/158 | 191/191 | 271/271 | 187/187 |
| In | Fall | 2019 |  |  | 234/234 |  |  | 271/271 | 187/187 |
| In | Fall | 2019 |  |  | 234/234 | 158/158 | 191/191 | 271/271 | 187/187 |
| In | Fall | 2019 |  |  | 234/234 |  |  | 271/271 | 187/187 |
| Out | Fall | 2019 | 294/294 | 319/319 | 234/234 | 158/158 | 182/182 | 271/271 | 187/187 |
| Out | Fall | 2019 | 294/294 | 319/319 | 237/237 | 158/158 | 191/191 | 271/271 | 187/187 |
| Out | Fall | 2019 | 294/294 | 325/325 |  | 158/158 |  | 271/271 | 187/187 |
| Out | Fall | 2019 | 294/294 | 319/319 | 234/234 | 158/158 | 182/182 | 271/271 | 187/187 |
| Out | Fall | 2019 | 294/294 | 325/325 |  | 158/158 | 182/182 | 271/271 | 187/187 |
| Out | Fall | 2019 | 294/294 | 319/319 | 234/234 | 158/158 | 182/191 | 271/271 | 187/187 |
| Out | Fall | 2019 | 294/294 | 319/319 | 234/234 | 158/158 | 191/191 | 271/271 | 187/187 |
| Out | Fall | 2019 | 294/294 | 319/319 | 234/234 | 158/158 | 158/158 | 271/271 | 187/187 |
| Out | Fall | 2019 | 294/294 | 319/319 | 234/234 | 158/158 | 182/182 | 271/271 | 187/187 |
| Out | Fall | 2019 | 294/294 | 319/319 | 234/234 |  | 182/182 | 271/271 | 187/187 |
| Out | Fall | 2019 | 294/294 | 319/319 | 234/234 | 158/158 | 191/191 | 271/271 | 187/187 |
| Out | Fall | 2019 | 294/294 | 319/319 | 234/234 | 158/158 | 191/191 | 271/271 | 187/187 |
| Out | Fall | 2019 | 294/294 | 319/319 | 234/234 | 158/158 | 182/182 | 271/271 | 187/187 |
| Out | Fall | 2019 | 294/294 | 319/319 | 234/234 | 158/158 | 158/158 | 271/271 | 187/187 |
| Pop | Season | Year | ALB139s | ALB139d | ALB158d | ALB165s | ALB165d | ALB230d | ALB256d |
| Out | Fall | 2019 |  |  | 234/234 | 158/158 | 191/191 | 271/271 | 187/187 |
| Out | Fall | 2019 |  |  | 234/234 | 158/158 | 191/191 | 271/271 | 187/187 |
| Out | Fall | 2019 |  |  | 234/234 | 158/158 | 191/191 | 271/271 | 187/187 |
| Out | Fall | 2019 |  |  | 234/234 | 158/158 | 191/191 | 271/271 | 187/187 |
| Out | Fall | 2019 |  |  | 234/234 | 158/158 | 182/182 | 271/271 | 187/187 |
| Out | Fall | 2019 |  |  | 234/234 | 158/158 | 182/182 | 271/271 | 187/187 |
| Out | Fall | 2019 |  |  | 234/234 | 158/158 | 182/182 | 271/271 | 187/187 |
| Out | Fall | 2019 |  |  | 234/234 | 158/158 | 182/182 | 271/271 | 187/187 |
| Out | Fall | 2019 |  |  |  | 158/158 | 182/182 | 271/271 |  |
| Out | Fall | 2019 |  |  |  | 158/158 | 191/191 | 271/271 |  |
| Out | Fall | 2019 |  |  |  | 158/158 | 191/191 | 271/271 |  |
| Out | Fall | 2019 |  |  | 234/234 | 158/158 | 182/182 | 271/271 | 187/187 |
| Out | Fall | 2019 |  |  | 234/234 | 158/158 | 182/191 | 271/271 | 187/187 |
| Out | Fall | 2019 |  |  | 234/234 | 158/158 | 191/191 | 271/271 | 187/187 |
| Out | Fall | 2019 |  |  | 234/234 | 158/158 | 158/158 | 271/271 | 187/187 |
| Out | Fall | 2019 |  |  | 234/234 | 158/158 | 182/182 | 271/271 | 187/187 |
| Out | Fall | 2019 |  |  | 234/234 |  | 182/182 | 271/271 | 187/187 |
| Out | Fall | 2019 |  |  | 234/234 | 158/158 | 191/191 | 271/271 | 187/187 |
| Out | Fall | 2019 |  |  | 234/234 | 158/158 | 191/191 | 271/271 | 187/187 |
| Out | Fall | 2019 |  |  | 234/234 | 158/158 | 182/182 | 271/271 | 187/187 |
| Out | Fall | 2019 |  |  |  | 158/158 | 158/158 | 271/271 | 187/187 |
| Out | Fall | 2019 |  |  | 234/234 | 158/158 | 182/191 | 271/271 | 187/187 |
| Out | Fall | 2019 |  |  | 234/234 | 158/158 | 191/191 | 271/271 | 187/187 |
| In | Spring | 2018 | 294/294 | 319/319 | 234/234 | 158/158 | 191/191 | 271/271 | 187/187 |
| In | Spring | 2018 | 294/294 | 319/319 | 234/234 | 158/158 | 191/191 | 271/271 | 187/187 |
| In | Spring | 2018 | 294/294 | 319/319 | 234/234 | 158/158 | 182/182 | 271/271 | 187/187 |
| In | Spring | 2018 | 294/294 | 319/319 | 234/234 | 158/158 | 191/191 | 271/271 | 187/187 |
| In | Spring | 2018 | 294/294 | 319/319 | 234/237 | 158/158 | 185/185 | 271/271 | 187/187 |
| In | Spring | 2018 | 294/294 | 319/319 | 234/237 | 158/158 | 191/191 | 271/271 | 187/187 |
| In | Spring | 2018 | 294/294 | 319/319 | 234/234 | 158/158 | 185/185 | 271/271 | 187/187 |
| In | Spring | 2018 | 294/294 | 319/319 | 237/237 | 158/158 | 182/182 | 271/271 | 188/188 |
| In | Spring | 2018 | 294/294 | 319/319 | 234/234 | 158/158 | 182/182 | 271/271 | 187/187 |
| In | Spring | 2018 | 294/294 | 319/319 | 234/234 | 158/158 | 191/191 | 271/271 | 187/187 |
| In | Spring | 2018 | 294/294 | 319/319 | 234/234 | 158/158 | 182/182 | 271/271 | 187/187 |
| In | Spring | 2018 | 294/294 | 319/319 |  | 158/158 | 182/182 | 271/271 | 187/187 |
| In | Spring | 2018 | 294/294 | 319/319 |  | 158/158 | 191/191 | 271/271 | 187/187 |
| In | Spring | 2018 | 294/294 | 319/319 |  | 158/158 | 191/191 | 271/271 | 187/187 |
| In | Spring | 2018 | 294/294 | 319/319 |  | 158/158 | 182/182 | 271/271 | 187/187 |
| In | Spring | 2018 | 294/294 | 319/319 |  | 158/158 | 185/185 | 271/271 | 187/187 |
| In | Spring | 2018 | 294/294 | 319/319 |  | 158/158 | 182/182 | 271/271 | 187/187 |
| In | Spring | 2018 | 294/294 | 319/319 |  | 158/158 | 185/185 | 271/271 |  |
| In | Spring | 2018 | 294/294 | 319/319 | 234/234 | 158/158 | 191/191 | 271/271 | 187/187 |
| In | Spring | 2018 | 294/294 | 319/319 | 234/234 | 158/158 | 182/182 | 271/271 | 187/187 |
| In | Spring | 2018 | 294/294 | 319/319 | 234/234 | 158/158 | 191/191 | 271/271 | 187/187 |
| In | Spring | 2018 | 294/294 | 319/319 | 234/234 | 158/158 | 182/182 | 271/271 | 187/187 |
| In | Spring | 2018 | 294/294 | 319/319 | 234/234 | 158/158 | 191/191 | 271/271 | 187/187 |
| In | Spring | 2018 | 294/294 | 319/319 | 234/234 | 158/158 | 191/191 | 271/271 | 187/187 |
| In | Spring | 2018 | 294/294 | 319/319 | 234/234 | 158/158 | 191/191 | 271/271 | 187/187 |
| In | Spring | 2018 | 294/294 | 319/319 | 234/234 | 158/158 | 191/191 | 271/271 | 187/187 |
| In | Spring | 2018 | 294/294 | 319/319 | 234/234 | 158/158 | 191/191 | 271/271 | 187/187 |
| In | Spring | 2018 | 294/294 | 319/319 | 234/234 | 158/158 | 191/191 | 271/271 | 187/187 |
| In | Spring | 2018 | 294/294 | 319/319 | 234/234 | 158/158 | 182/182 | 271/271 | 188/188 |
| In | Spring | 2018 | 294/294 | 319/319 | 234/234 | 158/158 | 191/191 | 271/271 | 187/187 |
| In | Spring | 2018 | 294/294 | 319/319 | 234/234 | 158/158 | 191/191 | 271/271 | 187/187 |
| In | Spring | 2018 | 294/294 | 319/319 | 234/234 | 158/158 | 191/191 | 271/271 | 187/187 |
| In | Spring | 2018 | 294/294 | 319/319 | 237/237 | 158/158 | 185/185 | 271/271 | 187/187 |
| In | Summer | 2018 | 294/294 | 319/319 | 234/234 | 158/158 | 182/182 | 271/271 | 187/187 |
| In | Summer | 2018 | 294/294 | 319/319 | 234/234 | 158/158 | 191/191 | 271/271 | 187/187 |
| In | Summer | 2018 | 294/294 | 319/319 | 234/234 | 158/158 | 191/191 | 271/271 | 187/187 |
| Pop | Season | Year | ALB139s | ALB139d | ALB158d | ALB165s | ALB165d | ALB230d | ALB256d |
| In | Summer | 2018 | 294/294 | 319/319 | 234/234 | 158/158 | 158/158 | 271/271 | 187/187 |
| In | Summer | 2018 | 294/294 | 337/337 | 234/234 | 158/158 | 191/191 | 271/271 | 187/187 |
| In | Summer | 2018 | 294/294 | 319/319 | 234/234 | 158/158 | 191/191 | 271/271 | 187/187 |
| Out | Spring | 2018 | 294/294 | 319/319 | 234/234 | 158/158 | 182/182 | 271/271 | 187/187 |
| Out | Spring | 2018 | 294/294 | 319/319 | 234/234 | 158/158 | 191/191 | 271/271 | 187/187 |
| Out | Spring | 2018 |  |  | 234/234 | 158/158 | 176/176 | 271/271 | 187/187 |
| Out | Spring | 2018 | 294/294 | 319/319 | 234/234 | 158/158 | 191/191 |  |  |
| Out | Spring | 2018 | 294/294 | 319/319 | 234/234 | 158/158 |  | 271/271 | 187/187 |
| Out | Spring | 2018 | 294/294 | 319/319 | 234/234 | 158/158 | 191/191 |  | 187/187 |
| Out | Spring | 2018 | 294/294 | 319/319 | 234/234 | 158/158 | 191/191 | 271/271 | 187/187 |
| Out | Spring | 2018 | 294/294 | 319/319 | 234/234 | 158/158 | 191/191 | 271/271 | 187/187 |
| Out | Spring | 2018 | 294/294 | 319/319 | 234/234 | 158/158 | 191/191 | 271/271 | 187/187 |
| Out | Spring | 2018 | 294/294 | 319/319 | 234/234 | 158/158 | 191/191 | 271/271 | 187/187 |
| Out | Spring | 2018 | 294/294 | 319/319 | 234/234 | 158/158 | 191/191 | 271/271 | 187/187 |
| Out | Spring | 2018 | 294/294 | 319/319 | 234/234 | 158/158 | 191/191 | 271/271 | 187/187 |
| Out | Spring | 2018 | 294/294 | 319/319 | 234/234 | 158/158 | 182/182 | 271/271 | 187/187 |
| Out | Spring | 2018 | 294/294 | 319/319 | 234/234 | 158/158 | 191/191 | 271/271 | 187/187 |
| Out | Spring | 2018 | 294/294 | 319/319 | 234/234 | 158/158 | 191/191 | 271/271 | 187/187 |
| Out | Spring | 2018 | 294/294 | 319/319 | 234/234 | 158/158 | 191/191 | 271/271 | 187/187 |
| Out | Summer | 2018 | 294/294 | 319/319 | 234/234 | 158/158 | 182/182 | 271/271 | 187/187 |
| Out | Summer | 2018 | 294/294 | 319/319 | 237/237 | 158/158 | 185/185 | 271/271 | 187/187 |
| Out | Summer | 2018 | 294/294 | 319/319 | 234/234 | 158/158 | 191/191 | 271/271 | 187/187 |
| Out | Summer | 2018 | 294/294 | 337/337 | 234/234 | 158/158 | 182/182 |  | 187/187 |
| Out | Summer | 2018 | 294/294 | 319/319 | 234/234 | 158/158 | 191/191 | 271/271 | 187/187 |
| Out | Summer | 2018 | 294/294 | 319/319 | 234/234 | 158/158 | 191/191 | 271/271 | 187/187 |
| Out | Spring | 2018 | 294/294 | 319/319 | 234/234 | 158/158 | 182/182 | 271/271 | 187/187 |
| Out | Spring | 2018 | 294/294 | 319/319 | 234/234 | 158/158 | 191/191 | 271/271 | 187/187 |
| Out | Spring | 2018 | 294/294 | 319/319 | 234/234 | 158/158 | 182/182 | 271/271 | 187/187 |
| Out | Spring | 2018 | 294/294 | 319/319 | 234/234 | 158/158 | 191/191 | 271/271 | 187/187 |
| Out | Spring | 2018 | 294/294 | 319/319 | 234/234 | 158/158 | 191/191 | 271/271 | 187/187 |
| Out | Summer | 2018 | 294/294 | 319/319 | 234/234 | 158/158 | 182/182 | 271/271 | 187/187 |
| Out | Summer | 2018 | 294/294 | 319/319 | 234/234 | 158/158 | 191/191 | 271/271 | 187/187 |
| Out | Summer | 2018 | 294/294 | 319/319 | 234/234 | 158/158 | 191/191 | 271/271 | 187/187 |
| Out | Spring | 2018 | 294/294 | 319/319 | 234/234 | 158/158 | 182/182 | 271/271 | 187/187 |
| Out | Spring | 2018 | 294/294 | 319/319 | 234/234 | 158/158 | 182/182 | 271/271 | 187/187 |
| Out | Spring | 2018 | 294/294 | 319/319 | 234/234 | 158/158 | 182/182 | 271/271 | 187/187 |
| Out | Spring | 2019 | 294/294 | 319/319 | 234/234 | 158/158 | 191/191 | 271/271 | 188/188 |
| Out | Spring | 2019 | 294/294 | 319/319 | 234/234 | 158/158 | 191/191 | 271/271 | 187/187 |
| Out | Spring | 2019 | 294/294 | 319/319 | 234/234 | 158/158 | 182/182 | 271/271 | 187/187 |
| Out | Spring | 2019 | 294/294 | 319/319 | 234/234 | 158/158 | 182/182 | 271/271 | 187/187 |
| Out | Spring | 2019 | 294/294 | 319/319 | 234/234 | 158/158 | 191/191 | 271/271 | 187/187 |
| Out | Spring | 2019 | 294/294 | 319/319 | 234/234 | 158/158 | 176/176 |  |  |
| Out | Spring | 2019 | 294/294 | 319/319 | 234/234 | 158/158 | 176/176 | 271/271 | 187/187 |
| Out | Spring | 2019 | 294/294 | 319/319 | 234/234 | 158/158 | 191/191 | 271/271 | 187/187 |
| Out | Spring | 2019 | 294/294 | 319/319 | 234/234 | 158/158 | 191/191 | 271/271 | 187/187 |
| Out | Spring | 2019 | 294/294 | 319/319 | 234/234 | 158/158 | 191/191 |  | 187/187 |
| Out | Spring | 2019 | 294/294 | 319/319 | 234/234 | 158/158 | 191/191 | 271/271 | 187/187 |
| Out | Spring | 2019 | 294/294 | 319/319 | 234/234 | 158/158 | 182/182 | 271/271 | 187/187 |
| Out | Spring | 2019 | 294/294 | 319/319 | 234/234 | 158/158 | 191/191 | 271/271 | 187/187 |
| Out | Spring | 2019 | 294/294 | 319/319 | 234/234 | 158/158 | 191/191 |  | 188/188 |
| Out | Spring | 2019 | 294/294 | 319/319 | 234/234 | 158/158 | 182/182 | 271/271 | 187/187 |
| Out | Spring | 2019 | 294/294 | 319/319 | 234/234 | 158/158 | 182/182 | 271/271 | 187/187 |
| Out | Spring | 2019 | 294/294 | 319/319 | 234/234 | 158/158 | 191/191 |  | 188/188 |
| Out | Spring | 2019 | 294/294 | 319/319 | 234/234 | 158/158 | 182/182 | 271/271 | 187/187 |

| Pop | Season | Year | ALB372d | ALB445d |
| --- | --- | --- | --- | --- |
| In | Fall | 2018 | 200/200 | 220/220 |
| In | Fall | 2018 | 200/200 | 220/220 |
| In | Fall | 2018 | 200/200 | 220/220 |
| In | Fall | 2018 | 200/200 | 220/220 |
| In | Fall | 2018 | 197/197 | 220/220 |
| In | Fall | 2018 | 179/179 | 220/220 |
| In | Fall | 2018 | 179/179 | 220/220 |
| In | Fall | 2018 | 200/200 | 220/220 |
| In | Fall | 2018 | 179/179 | 220/220 |
| In | Fall | 2018 | 179/189 | 220/220 |
| In | Fall | 2018 | 185/185 | 220/220 |
| In | Fall | 2019 | 200/200 | 220/220 |
| In | Fall | 2019 | 179/200 | 220/220 |
| In | Fall | 2019 | 179/179 | 220/220 |
| In | Fall | 2019 | 179/179 | 220/220 |
| In | Fall | 2019 | 200/200 | 220/220 |
| In | Fall | 2019 | 179/179 | 220/220 |
| In | Fall | 2019 | 197/197 | 220/220 |
| In | Fall | 2019 | 179/179 | 220/220 |
| In | Fall | 2019 | 179/179 | 220/220 |
| In | Fall | 2019 | 179/179 | 220/220 |
| In | Fall | 2019 | 179/179 | 220/220 |
| In | Fall | 2019 | 179/179 | 220/220 |
| In | Fall | 2019 | 179/179 | 220/220 |
| In | Fall | 2019 | 200/200 | 220/220 |
| In | Fall | 2019 | 179/179 | 220/220 |
| In | Fall | 2019 | 179/179 | 220/220 |
| In | Fall | 2019 | 200/200 | 220/220 |
| In | Fall | 2019 | 189/189 | 205/205 |
| In | Fall | 2019 | 179/179 | 220/220 |
| In | Fall | 2019 | 179/179 | 220/220 |
| In | Fall | 2019 | 179/179 | 205/205 |
| In | Fall | 2019 | 179/179 | 220/220 |
| In | Fall | 2019 | 179/179 | 205/205 |
| In | Fall | 2019 | 179/179 | 220/220 |
| In | Fall | 2019 | 179/179 | 220/220 |
| In | Fall | 2019 | 179/179 | 220/220 |
| In | Fall | 2019 | 179/179 | 220/220 |
| In | Fall | 2019 | 179/179 | 220/220 |
| In | Fall | 2019 | 197/197 | 205/205 |
| In | Fall | 2019 | 179/179 | 220/220 |
| In | Fall | 2019 | 179/179 | 220/220 |
| In | Fall | 2019 | 179/179 | 220/220 |
| In | Fall | 2019 | 179/179 | 220/220 |
| Out | Fall | 2019 | 200/200 | 220/220 |
| Out | Fall | 2019 | 179/179 | 220/220 |
| Out | Fall | 2019 | 182/182 | 244/244 |
| Out | Fall | 2019 | 179/179 | 205/205 |
| Out | Fall | 2019 | 182/182 | 244/244 |
| Out | Fall | 2019 | 200/200 | 205/205 |
| Out | Fall | 2019 | 179/179 | 220/220 |
| Out | Fall | 2019 | 200/200 | 205/205 |
| Out | Fall | 2019 | 179/179 | 220/220 |
| Out | Fall | 2019 | 179/179 | 220/220 |
| Out | Fall | 2019 | 179/179 | 220/220 |
| Out | Fall | 2019 | 179/179 | 220/220 |
| Out | Fall | 2019 | 200/200 | 220/220 |
| Out | Fall | 2019 | 200/200 | 220/220 |
| Pop | Season | Year | ALB372d | ALB445d |
| Out | Fall | 2019 | 179/179 | 220/220 |
| Out | Fall | 2019 | 179/200 | 220/220 |
| Out | Fall | 2019 | 179/179 | 220/220 |
| Out | Fall | 2019 | 179/179 | 220/220 |
| Out | Fall | 2019 | 179/179 | 220/220 |
| Out | Fall | 2019 | 200/200 | 220/220 |
| Out | Fall | 2019 | 179/179 | 220/220 |
| Out | Fall | 2019 | 200/200 | 205/205 |
| Out | Fall | 2019 | 189/189 | 205/205 |
| Out | Fall | 2019 | 189/189 | 205/205 |
| Out | Fall | 2019 | 189/189 | 205/205 |
| Out | Fall | 2019 | 179/200 | 205/205 |
| Out | Fall | 2019 | 200/200 | 220/220 |
| Out | Fall | 2019 | 200/200 | 205/205 |
| Out | Fall | 2019 | 200/200 | 220/220 |
| Out | Fall | 2019 | 200/200 | 220/220 |
| Out | Fall | 2019 | 200/200 | 205/205 |
| Out | Fall | 2019 | 179/179 | 220/220 |
| Out | Fall | 2019 | 179/179 | 220/220 |
| Out | Fall | 2019 | 200/200 | 220/220 |
| Out | Fall | 2019 | 182/182 | 205/205 |
| Out | Fall | 2019 | 179/200 | 220/220 |
| Out | Fall | 2019 | 179/179 | 220/220 |
| In | Spring | 2018 | 179/179 | 220/220 |
| In | Spring | 2018 | 179/179 | 220/220 |
| In | Spring | 2018 | 200/200 | 220/220 |
| In | Spring | 2018 | 179/179 | 220/220 |
| In | Spring | 2018 | 179/179 | 220/220 |
| In | Spring | 2018 | 179/179 | 220/220 |
| In | Spring | 2018 | 179/179 | 220/220 |
| In | Spring | 2018 | 197/197 | 220/220 |
| In | Spring | 2018 | 179/179 | 205/205 |
| In | Spring | 2018 | 179/179 | 220/220 |
| In | Spring | 2018 | 200/200 | 220/220 |
| In | Spring | 2018 | 200/200 | 220/220 |
| In | Spring | 2018 | 179/179 | 220/220 |
| In | Spring | 2018 | 179/179 | 220/220 |
| In | Spring | 2018 | 200/200 | 220/220 |
| In | Spring | 2018 | 179/179 | 220/220 |
| In | Spring | 2018 | 179/179 | 205/205 |
| In | Spring | 2018 | 179/179 | 220/220 |
| In | Spring | 2018 | 179/179 | 220/220 |
| In | Spring | 2018 | 200/200 | 220/220 |
| In | Spring | 2018 | 179/179 | 220/220 |
| In | Spring | 2018 | 200/200 | 220/220 |
| In | Spring | 2018 | 179/179 | 220/220 |
| In | Spring | 2018 | 179/179 | 220/220 |
| In | Spring | 2018 | 179/179 | 220/220 |
| In | Spring | 2018 | 179/179 | 220/220 |
| In | Spring | 2018 | 179/179 | 220/220 |
| In | Spring | 2018 | 179/179 | 220/220 |
| In | Spring | 2018 | 179/179 | 220/220 |
| In | Spring | 2018 | 179/179 | 220/220 |
| In | Spring | 2018 | 179/179 | 220/220 |
| In | Spring | 2018 | 179/179 | 220/220 |
| In | Spring | 2018 | 179/179 | 220/220 |
| In | Summer | 2018 | 179/179 | 220/220 |
| In | Summer | 2018 | 200/200 | 220/220 |
| In | Summer | 2018 | 179/179 | 220/220 |
| Pop | Season | Year | ALB372d | ALB445d |
| In | Summer | 2018 | 179/179 | 220/220 |
| In | Summer | 2018 | 197/197 | 220/220 |
| In | Summer | 2018 | 179/179 | 220/220 |
| Out | Spring | 2018 | 200/200 | 220/220 |
| Out | Spring | 2018 | 179/179 | 220/220 |
| Out | Spring | 2018 |  | 205/220 |
| Out | Spring | 2018 | 179/179 | 220/220 |
| Out | Spring | 2018 |  | 205/220 |
| Out | Spring | 2018 | 179/179 | 220/220 |
| Out | Spring | 2018 | 179/179 | 220/220 |
| Out | Spring | 2018 | 179/179 | 220/220 |
| Out | Spring | 2018 | 179/179 |  |
| Out | Spring | 2018 | 179/179 | 220/220 |
| Out | Spring | 2018 | 179/179 | 220/220 |
| Out | Spring | 2018 | 179/179 | 220/220 |
| Out | Spring | 2018 | 179/179 | 205/205 |
| Out | Spring | 2018 | 179/179 | 220/220 |
| Out | Spring | 2018 | 179/179 | 220/220 |
| Out | Spring | 2018 | 179/179 | 220/220 |
| Out | Summer | 2018 | 200/200 | 220/220 |
| Out | Summer | 2018 | 179/179 | 220/220 |
| Out | Summer | 2018 | 179/179 | 220/220 |
| Out | Summer | 2018 | 197/197 | 220/220 |
| Out | Summer | 2018 | 179/179 | 220/220 |
| Out | Summer | 2018 | 179/179 | 220/220 |
| Out | Spring | 2018 | 200/200 | 220/220 |
| Out | Spring | 2018 | 179/179 | 220/220 |
| Out | Spring | 2018 | 200/200 | 220/220 |
| Out | Spring | 2018 | 179/179 | 220/220 |
| Out | Spring | 2018 | 179/179 | 220/220 |
| Out | Summer | 2018 | 179/179 | 220/220 |
| Out | Summer | 2018 | 179/179 | 220/220 |
| Out | Summer | 2018 | 179/179 | 220/220 |
| Out | Spring | 2018 |  | 220/220 |
| Out | Spring | 2018 | 200/200 | 220/220 |
| Out | Spring | 2018 | 200/200 | 220/220 |
| Out | Spring | 2019 | 179/179 | 220/220 |
| Out | Spring | 2019 | 179/179 | 220/220 |
| Out | Spring | 2019 | 200/200 | 220/220 |
| Out | Spring | 2019 | 200/200 | 220/220 |
| Out | Spring | 2019 | 179/179 | 220/220 |
| Out | Spring | 2019 | 179/179 | 220/220 |
| Out | Spring | 2019 | 179/179 | 220/220 |
| Out | Spring | 2019 | 179/179 | 220/220 |
| Out | Spring | 2019 | 179/179 | 220/220 |
| Out | Spring | 2019 | 179/179 | 220/220 |
| Out | Spring | 2019 |  | 220/220 |
| Out | Spring | 2019 | 200/200 | 220/220 |
| Out | Spring | 2019 | 179/179 | 220/220 |
| Out | Spring | 2019 | 179/179 | 220/220 |
| Out | Spring | 2019 | 200/200 | 220/220 |
| Out | Spring | 2019 | 179/179 | 205/205 |
| Out | Spring | 2019 | 179/179 | 220/220 |
| Out | Spring | 2019 | 200/200 | 205/205 |
