# Supplementary material for: The genetic structure of a Brachypodium hybridum population in a patchy arid landscape is independent of neighboring perennials and stable over two consecutive years
Source: PeerJ. 2026 Mar 2;14:e20787. doi: 10.7717/peerj.20787 (PMC12962130; doi:10.7717/peerj.20787)
Supplement: Supplemental Information 3 — AMOVA showing partitioning of genetic variation between canopy locations (In vs Out), among individuals within locations, and within individuals. Reported columns: degrees of freedom (df), sum of squares (SS), estimated variance component (σ), percent of total variation (%), Φ-statistics for each hierarchical comparison. P-values based on 999 permutations; *** denotes p < 0.001. [file peerj-14-20787-s003.docx]

|  |  |  |  |  |  |  |
| --- | --- | --- | --- | --- | --- | --- |
| **Source of Variation** | **df** | **Sum of squares** | **Variance (σ)** | **Variation (%)** | **Φ-statistic** | ***p*-value** |
| Between locations (In vs Out) | 1 | 12.38 | 0.027 | 0.57 | 0.0057 | 0.220 |
| Between individuals within location | 113 | 1048.72 | 4.575 | 96.67 | 0.9723 | < 0.001*** |
| Within individuals | 115 | 15 | 0.130 | 2.76 | 0.9724 | < 0.001*** |
| Total | 229 | 1076.10 | --- | 100 | --- | --- |
| **Sampling data (2018 vs 2019)** |  |  |  |  |  |  |
| Between years (2018 vs 2019) | 1 | 8.34 | -0.008 | -0.18 | 0.0018 | 0.441 |
| Between individuals within year | 113 | 1052.75 | 4.59 | 97.41 | 0.9724 | < 0.001*** |
| Within individuals | 115 | 15 | 0.13 | 2.77 | 0.9723 | < 0.001*** |
| Total | 229 | 1076.09 | --- | 100 | --- | --- |
