# Supplementary figures and images for: The genetic structure of a Brachypodium hybridum population in a patchy arid landscape is independent of neighboring perennials and stable over two consecutive years
Source: PeerJ. 2026 Mar 2;14:e20787. doi: 10.7717/peerj.20787 (PMC12962130; doi:10.7717/peerj.20787)

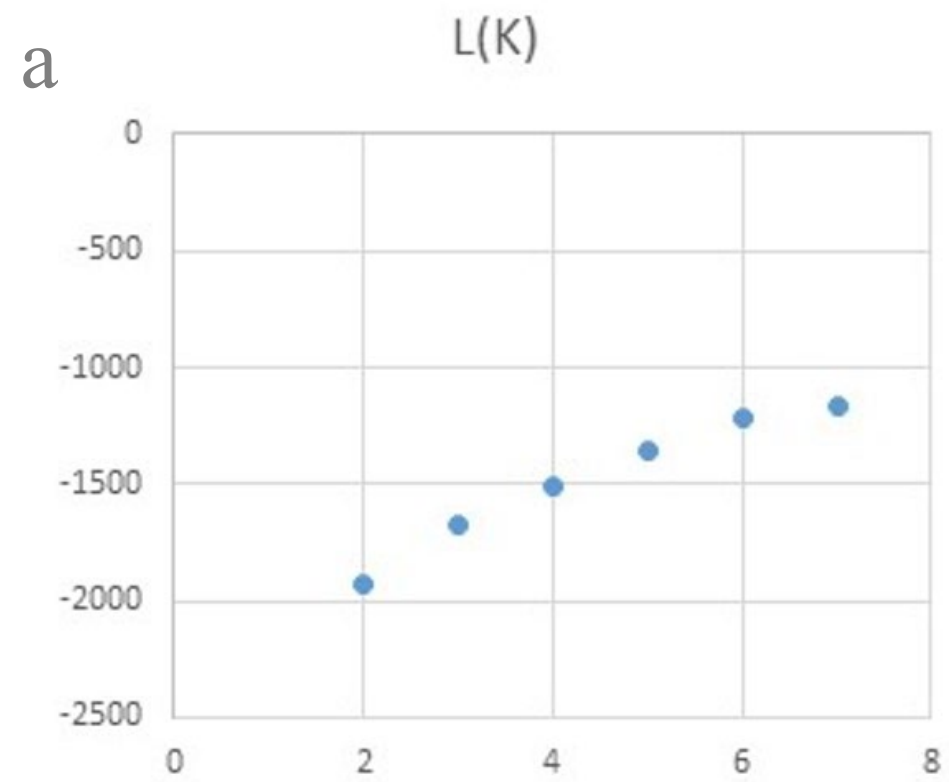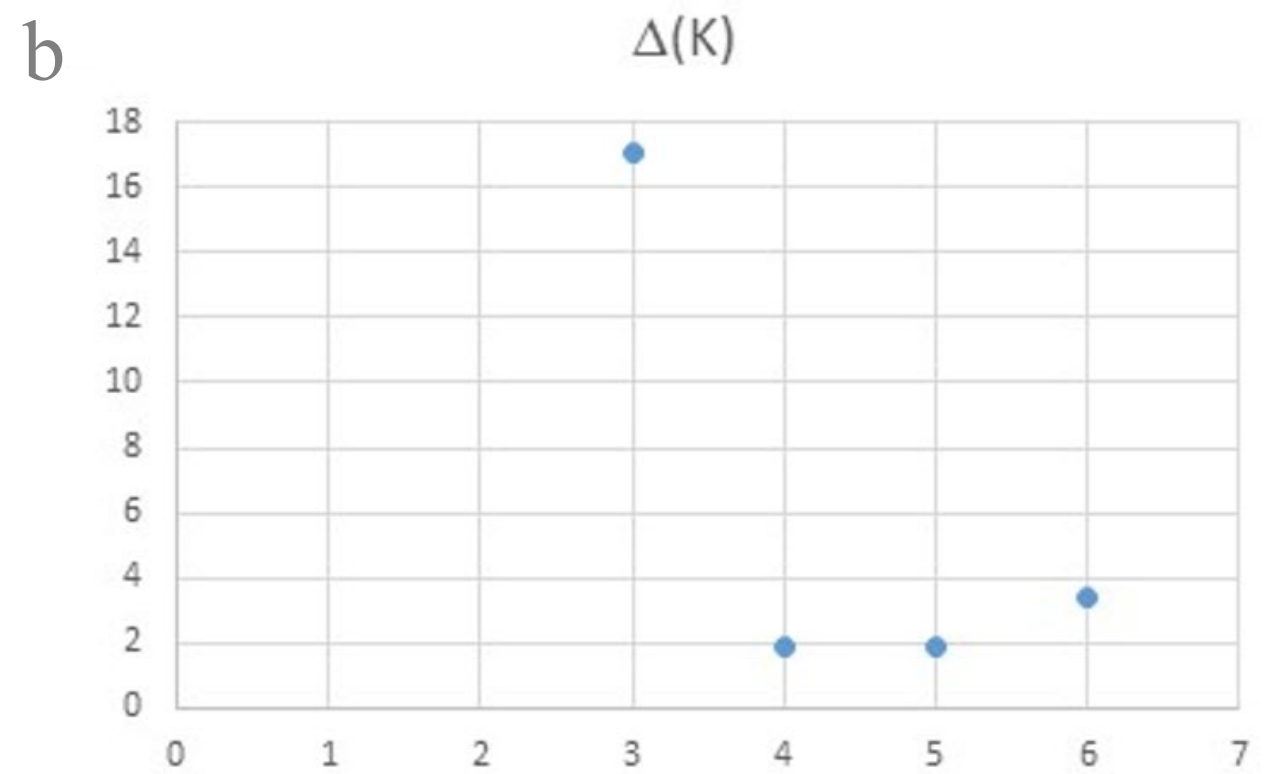

Supplement: Supplemental Information 4 [file peerj-14-20787-s004.pdf]
